# Thermal monitoring during photothermia: hybrid probes for simultaneous plasmonic heating and near-infrared optical nanothermometry

**DOI:** 10.7150/thno.38091

**Published:** 2019-09-25

**Authors:** Marta Quintanilla, Isabel García, Irene de Lázaro, Rafaela García-Alvarez, Malou Henriksen-Lacey, Sandra Vranic, Kostas Kostarelos, Luis M. Liz-Marzán

**Affiliations:** 1 CIC biomaGUNE and CIBER-BBN, Paseo de Miramón 182, 20014 Donostia-San Sebastián, Spain; 2 Nanomedicine Lab, Faculty of Biology, Medicine & Health and National Graphene Institute, The University of Manchester, AV Hill Building, Manchester M13 9PT, UK; 3 Ikerbasque, Basque Foundation for Science, 48013 Bilbao, Spain

**Keywords:** photothermal therapy, nanothermometry, plasmonic heating, brain cancer, luminescence sensing, near-infrared

## Abstract

The control of temperature during photothermal therapy is key to preventing unwanted damage in surrounding tissue or post-treatment inflammatory responses. Lack of accurate thermal control is indeed one of the main limitations that hyperthermia techniques present to allow their translation into therapeutic applications. We developed a nanoprobe that allows controlled local heating, combined with* in situ* nanothermometry. The design of the probe follows a practical rationale that aims at simplifying experimental requirements and exploits exclusively optical wavelengths matching the first and second biological windows in the near-infrared.

**Methods:** Hybrid nanostructures were chemically synthesized, and combine gold nanostars (photothermal agents) with CaF_2_:Nd^3+^,Y^3+^ nanoparticles (luminescent nanothermometers). Both components were simultaneously excited in the near-infrared range, at 808 nm. Following the goal of simplifying the thermal monitoring technique, the luminescent signal was recorded with a portable near-infrared detector. The performance of the probes was tested in 3D tumor spheroids from a human glioblastoma (U87MG) cell line. The location of the beads within the spheroids was determined measuring Nd^3+^ emission in a commercial Lightsheet microscope, modified in-house to be able to select the required near-infrared wavelengths. The temperature achieved inside the tumor spheroids was deduced from the luminescence of Nd^3+^, following a protocol that we developed to provide reliable thermal readings.

**Results:** The choice of materials was shown to work as an optically excited hybrid probe. Depending on the illumination parameters, temperature can be controlled in a range between 37 ºC and 100 ºC. The near-infrared emission of nanothermometers also allows microscopic tracking of the hybrid nanostructures, confirming that the probes can penetrate deeper into the spheroid mass. We observed that, application of optical thermometry in biological environments requires often neglected considerations, since the optical signal changes along the optical path. Accordingly, we developed data analysis protocols that guarantee reliable thermal readings.

**Conclusions:** The prepared hybrid probes are internalized in 3D tumor spheroids and can be used to induce cell death through photothermal effects, while simultaneously measuring the local temperature* in situ*. We show that luminescent thermometry in biomedical applications requires the development of protocols that guarantee accurate readings. Regarding photothermal treatments, we observe a sharp thermal threshold at around 55 ºC (for 10 min treatments) that separates high survival ratio from complete cell death.

## Introduction

Cells and most bacteria can only live and grow within a limited temperature range, often around 37 ºC. Differences of just a few degrees can readily lead to metabolic changes. Indeed, evolution in animals has shaped mechanisms to increase body-temperature by 1 to 4 ºC in response to inflammatory situations. This is manifested as high body temperature ('fever' in warm-blooded species) and has been shown to help increase survival in certain pathological conditions 1. Indeed, when artificially triggered, this mild level of heating has been exploited as an adjuvant setting to improve the success of certain cancer treatment strategies 1, 2. If elevated temperatures are achieved locally, cells start to get damaged, mainly due to protein denaturation 3. This limitation in the survival thermal range can be seen as an opportunity when it comes to developing medical therapies that require killing biological entities such as cancer cells or even bacteria. Such treatments are known as hyperthermal, and the elevated temperatures can be set above 45 ºC, whereas ablation therapy would require temperatures above 70 ºC.

Hyperthermia can be pursued in different ways to achieve a therapeutic effect. Particularly seeking a treatment against cancer, early assays in the late XIX century studied the administration of bacteria to create an infection that would naturally trigger a fever response 4. Most current options emerged following the synthesis of nanomaterials that can transform an external stimulus into heat and can be selectively placed in the tumor area. Particles made of those materials appear as particularly suitable candidates to trigger localized heating, even in deep tissue if the stimulus can penetrate through the body. The hyperthermia technique was first demonstrated using magnetic nanoparticles, which would release heat upon application of an alternating magnetic field 5. More recently, nanoparticles that release heat under optical excitation have also been developed, leading to so-called photothermal therapy. This raised an ongoing debate on the suitability of each type of thermal therapy depending on the disease (on tumor location, for instance) 6. Although photothermal nanoparticles can be made of a wide variety of materials, including quantum dots, lanthanide-doped nanoparticles or carbon dots, the highest heating efficiencies have been achieved by using gold-based nanoparticles 2, 7.

In all cases, due to the rather uncontrolled distribution of the nanoparticles within biological tissue and the heterogeneity of this environment, it is hard to achieve an accurate control of the temperature reached during the treatment. Actual thermal information of the treated area is required to design successful treatments that can kill the tumors while preserving the surrounding environment. This is especially important when one considers the two main mechanisms of cell death, apoptosis and necrosis, which can be tuned through small temperature changes 8. It is however key, to control the precise cell death mechanism, because it strongly affects the way the body reacts to the treatment, as e.g. necrosis may trigger inflammatory processes 9. Indeed, based on these considerations, lack of temperature control has been quoted as the main limitation of current hyperthermal probes 2, 10, 11.

Various techniques have been recently developed to measure temperature within micrometer sized areas 12, 13. However, measuring temperature during hyperthermal treatment holds strong limitations regarding the selection of thermometry techniques that can be applied. First of all, non-contact techniques should be used, aiming for a minimally invasive therapy. Within this category, the available strategies that can work in the complex biological environment are mainly based on luminescent molecules or nanoparticles 12. Second, not every luminescent thermometer would work, as it must be specific for temperature (independent of pH, composition of the environment, etc.) and both excitation and emission wavelengths should fall within the biological transparency windows, where absorption and scattering of light are minimized (first window: 750-950 nm; second window: 1000-1350 nm; third window: 1500-1700 nm) 14. A suitable selection of excitation and emission wavelengths is therefore of utmost importance 15. On one hand, scattering compromises the directionality of the excitation light through tissue; on the other hand, absorbance reduces the excitation power and produces unwanted heating along the light path. Moreover, commonly used short wavelength light (UV or blue) is known to have phototoxic effects, which is another good reason to move towards longer wavelengths 16, 17.

The combination of heating and temperature monitoring using nanoparticles is an ambitious goal that aims at extending and facilitating the application of hyperthermal therapy. Few attempts have been made to carry out hyperthermia with materials capable of both heating and emitting a temperature-dependent signal. This is the case for Co/Au and Fe/Au nanodomes, in which temperature is tracked optically through the viscosity-dependent rotation dynamics of the particles in the presence of a weak external AC magnetic field 18. Also, nanoparticles heavily doped with Nd^3+^ or some types of quantum dots have been explored 19-22. This approach benefits from the simplicity and robustness of the multifunctional probes, but given their limited heating efficiency (e.g., compared to gold nanoparticles) they require higher illumination doses to achieve a target temperature. Alternatively, hybrid structures have been proposed, by adding a thermometric capability to the most efficient hyperthermal probes 23-27. These approaches constitute interesting proof-of-concept experiments, but are limited in their biological applications as none of them fulfills the above listed requisites, including the use of near-IR wavelengths, validity in heterogeneous environments, and often involve complex detection or illumination systems requiring more than one light source.

We present here a hybrid nanostructure that combines plasmonic gold nanoparticles as an efficient optical heater, with a specifically designed nanothermometer based on CaF_2_:Nd^3+^,Y^3+^, which could be developed for *in vivo* applications. Both materials can be excited at the same wavelength, 808 nm, in the first biological window, while the nanothermometer emission is located within the second biological window. Accordingly, the luminescence signal from our nanothermometers also allows tracking and localization of the hybrid structure inside a biological tissue mass. We demonstrate application of the hybrid assemblies by photothermal killing of cancer cells, while recording temperature *in situ*, so that we can determine the thermal resolution they can achieve and define experimental protocols that provide an accurate thermal reading.

## Results and Discussion

**Figure 1:**
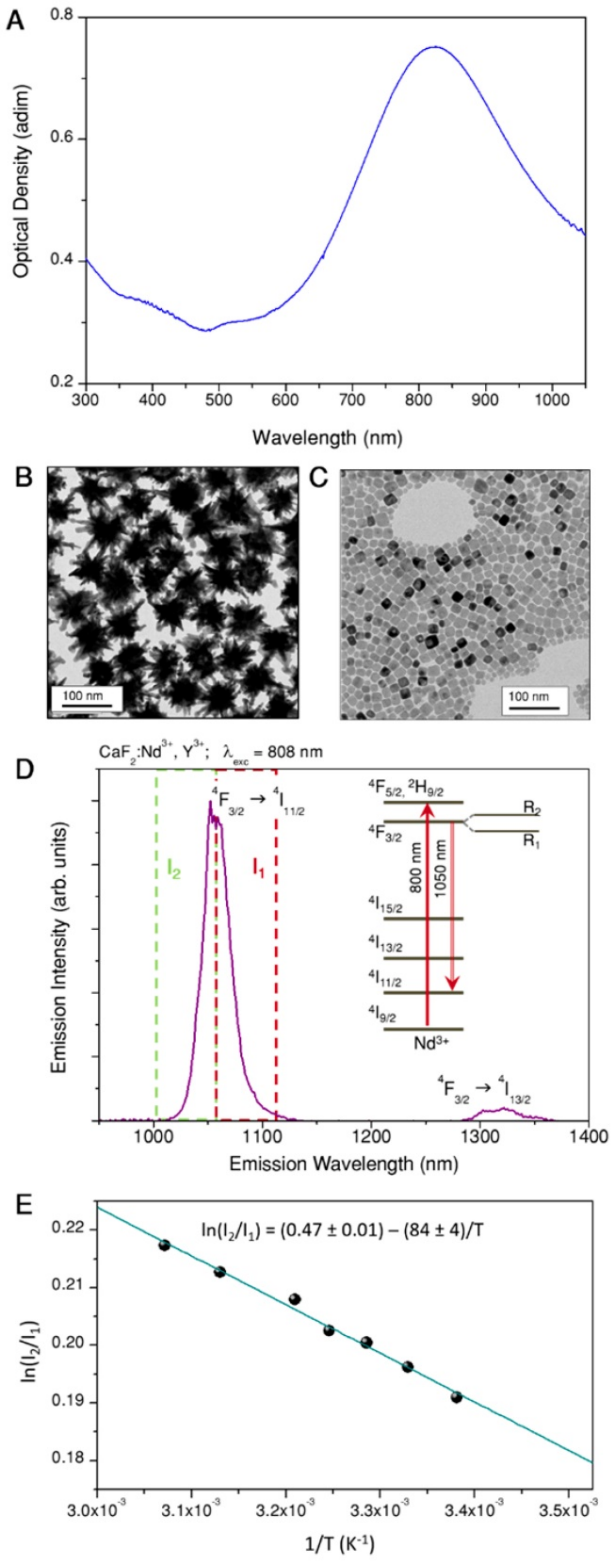
A) Extinction spectrum of gold nanostars. B) Representative TEM image of gold nanostars. C) Representative TEM image of CaF_2_:Nd^3+^,Y^3+^ nanoparticles. D) Emission spectrum under 808 nm excitation of CaF_2_:Nd^3+^,Y^3+^ nanoparticles, used as thermometers; a partial scheme of Nd^3+^ energy states is included in the inset; green and red dashed lines frame the two emission ranges used for ratiometric thermometry. E) Thermal calibration curve obtained by dividing the area covered by the emission intensities in the two highlighted regions in (D).

The selection and measurement of temperature in localized spots is key to controlling photothermal therapy and regulating irradiation doses for each specific treatment. The preparation of a probe that can be optically activated for both heating and temperature monitoring, requires a careful selection of materials for each specific task. Gold nanoparticles were chosen to play the role of heaters, since they are among the materials that most efficiently transform light into heat, while presenting negligible toxicity for human cells 28. The optimal illumination wavelength for heat production, as well as the heating efficiency, are largely determined by the nanoparticles morphology. Particularly efficient nanostructures comprise anisotropic shapes with sharp edges or pointy features, such as nanotriangles, nanorods or nanostars 29, 30. On the basis of prior experience, we decided to work with gold nanostars that are resonant at ca. 800 nm, as shown in Figure 1A.

Gold nanostars were prepared by seeded growth, using ascorbic acid as reducing agent and silver nitrate to assist the growth of spikes 31. The selected particles featured a tip-to-tip diameter of 83 ± 7 nm (See Figure 1B). Colloidal and morphological stability, as well as negative surface charge (required for subsequent fabrication steps), were achieved by surface functionalization with a thiolated polyethylene glycol (PEG), terminated in a carboxyl group. The heating efficiency of our gold nanostars at the laser wavelength used in photothermal experiments, was calculated to be 74% (For details on these measurements, refer to Section A1 and Figure S1 of the SI). Using the same excitation wavelength, heating efficiencies between 40% and 100% have been measured for gold nanostars of different sizes 29, which leaves those prepared here in the middle of that range.

To select our thermometric material, we placed emphasis on the requirement of working within the biological transparency windows, but also on the spectral compatibility with the heaters, the accuracy of the thermal reading and the possibility of retrieving absolute temperature values. Accordingly, we chose CaF_2_ nanoparticles doped with neodymium and yttrium as thermometers, which can be readily synthesized following a recently reported hydrothermal method 32, yielding cubic particles with 11 ± 3 nm side length (Figure 1C). As prepared, the particles are protected by citrate, and hence stable in aqueous solution. In CaF_2_:Nd^3+^,Y^3+^ particles, Nd^3+^ is the active ion absorbing light (at 808 nm) and featuring a strong emission band at 1050 nm (as shown in Figure 1D), whereas Y^3+^ is added to break energy migration paths between Nd^3+^ ions, which would otherwise quench the emission intensity. Following a previously reported discussion on this matter 33, we selected doping concentrations of [Nd^3+^] = 1%, [Y^3+^] = 15%. These nanoparticles can be excited at the same wavelength as our gold nanostars (see the energy diagram scheme in Figure 1D). Thus, both pursued functionalities, heating and thermal monitoring, can be triggered using a single light source. It is also noteworthy that lanthanide ions as dopants typically present characteristic narrow emission lines, which are easy to differentiate from any other signal from organic molecules present in biological tissues. The lanthanide emission additionally features a large Stokes shift, thereby minimizing the absorption of emitted light by nearby gold nanostars. These nanoparticles constitute a ratiometric thermometer based on the emission peak at 1050 nm. When Nd^3+^ ions are embedded inside a crystal, the electromagnetic field created by the surrounding atoms breaks the degeneracy of the ^4^F_3/2_ state (see scheme in Figure 1D), which gets divided into two sublevels, R_1_ and R_2_. Consequently, the emission at 1050 nm is actually an overlap of the emissions from these two sublevels. Given the small energy gap between R_1_ and R_2_ their electronic populations, and hence their emissions, are thermally linked. If we call I_1_ the emission intensity from R_1_, and I_2_ the emission intensity from R_2_, since the ratio between both intensities is given by a Boltzmann distribution, it can be written as:


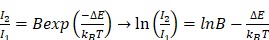
(1)

where B is an experimental constant, k_B_ is Boltzmann's constant and ΔE is the energy gap between R_1_ and R_2_. By applying logarithms to both sides of the equation, the relationship on the right is obtained, which allows us to plot the intensity ratio versus de inverse of temperature in the form of a linear function with slope -ΔE/k_B_. The main advantage of this type of thermometers is that the ratiometric technique is in itself a self-referenced method, thus providing absolute temperature measurements. Besides, the intensity ratio does not depend on the excitation power or on the concentration of particles, thereby minimizing the sources of error during thermal reading.

In Figure 1D we marked with two dashed squares the part of the spectrum to be integrated to retrieve I_1_ (red) and I_2_ (green), the limiting wavelength being 1057 nm 33. Thermal calibration can be performed by measuring the emission from a colloidal dispersion of the particles at various set temperatures. It is important for the accuracy of the calibration to use a low laser power, so as to minimize uncontrolled optical heating of the environment. We thereby obtain a plot such as that shown in Figure 1E. Following eq. (1) we can get a linear fit (blue line and equation within the graph), yielding a value of ΔE equal to 58 cm^-1^, which is consistent with previously reported values for the same doping concentration 33. Once the precise relationship between intensity ratio and temperature has been established for our nanoparticles, the opposite experiment can be performed to calculate temperature values from emission spectra.

We therefore prepared gold nanostars that could heat their nearby environment, as well as CaF_2_:Nd^3+^,Y^3+^ nanoparticles to measure temperature, both upon excitation at 808 nm. Co-localization of the components for photothermal experiments was ensured by co-assembly into hybrid structures. It should be noted that both nanostars and nanothermometers carry a negative surface charge, due to the selection of surfactants (carboxyl terminated PEG for nanostars, featuring a zeta potential of -25 mV, and citrate for nanothermometers, with a zeta potential of -7 mV), so they can be electrostatically linked onto positively charged surfaces. As a proof-of-concept, we chose polystyrene beads as a colloidal support 34. The selected beads had an average diameter of 500 nm and were functionalized with amino groups, thereby featuring a positive surface charge, indicated by a zeta potential value around +40 mV (Figure S2).

**Figure 2:**
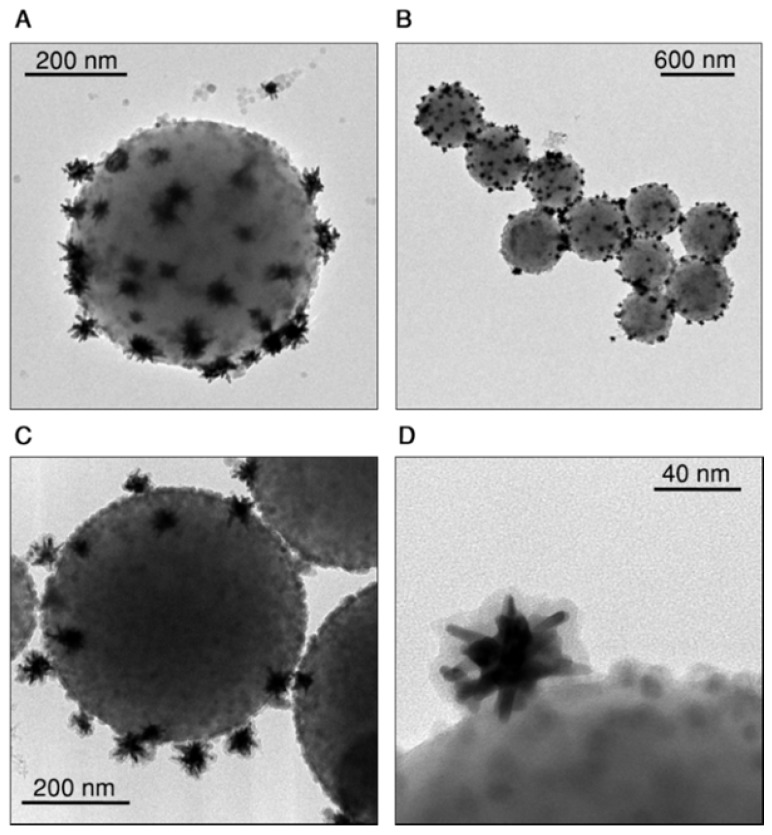
Representative TEM images of assembled beads before (A, B) and after (C, D) silica coating.

Upon electrostatic self-assembly by subsequent addition of CaF_2_ nanoparticles and gold nanostars to a dilute dispersion of beads, hybrid nanostructures were obtained as shown in Figure 2A and B (supplementary images are provided in Figures S3 and S4 of the SI). The gradual modification of the colloidal beads was confirmed by monitoring changes in zeta potential, which became negative (-18 mV) upon addition of the nanoparticles (Figure S2) and in the hydrodynamic diameter, which shifts from 515±6 nm to 750±20 nm (Figure S2). The TEM images show few free nanoparticles (gold nanostars and CaF_2_) next to the assemblies, even after four washing centrifugation steps. We concluded that these free particles likely derive from sample preparation for TEM (see short discussion in the SI, Figure S6). To prevent potential disassembly, which may also occur when the sample is placed in biological environments, we coated the hybrid probes with a thin shell of silica. We employed the standard Stöber method by adding tetraethyl orthosilicate (TEOS) to a dispersion of beads in a water/isopropanol mixture (H_2_O:IPA = 1:1.8) containing ammonia at pH 9 35, 36. By TEM analysis we measured a silica shell thickness around 7 nm, and confirmed homogenous coating of both gold nanostars and CaF_2_ nanoparticles on the beads surface (Figure 2C,D, and S7, SI). The hydrodynamic diameter increased up to 830 ± 30 nm as observed in DLS, with a further shift toward negative zeta potential, down to -40 mV (Figure S2). After silica coating, no free nanoparticles were found in TEM images (Figures 2C,D and S7, SI).

**Figure 3:**
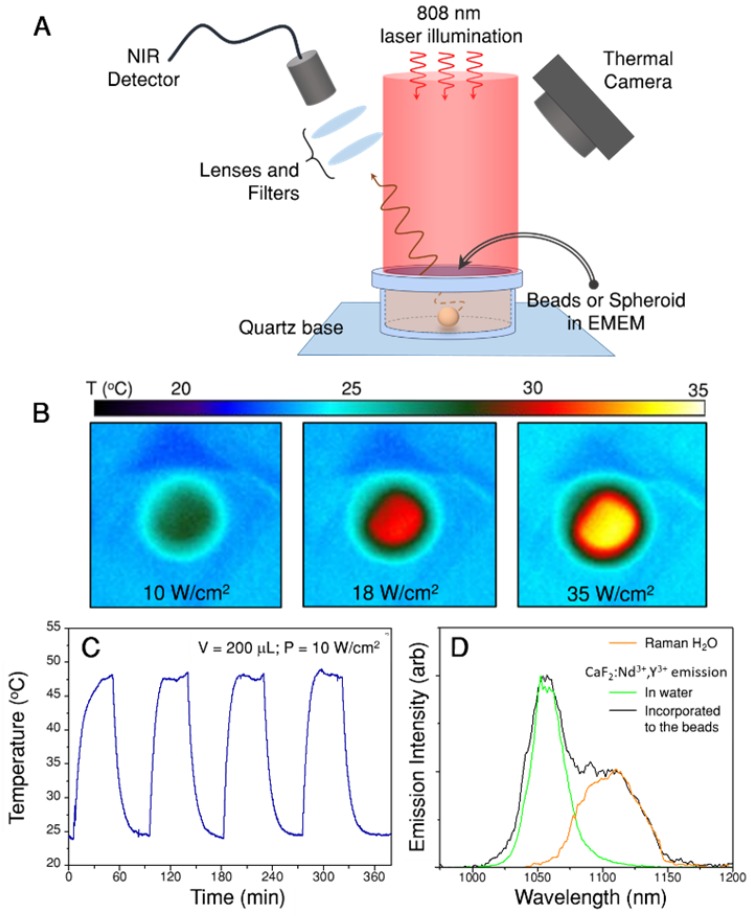
Heating and emitting properties of hybrid beads containing Au nanostars and CaF_2_:Nd^3+^_,_Y^3+^ nanoparticles. A) Experimental set-up used to illuminate the samples and record their luminescence while monitoring the temperature of the solution with a thermal camera. B) Thermal camera images upon illumination of 1 mL of beads solution (0.02 wt.%) with three different powers. C) Heating and cooling cycles of 200 µL (0.1 wt.%) in cell medium, EMEM. D) Emission spectrum recorded in the emission range of Nd^3+^ upon 808 nm illumination of a 200 µL dispersion in EMEM. The spectrum shows the expected emission of Nd^3+^ and an additional contribution from Raman scattering by water molecules.

The emitting and heating properties of the assemblies were tested in an aqueous dispersion (1 mL, 0.02 wt.%) homogeneously illuminated with an 808 nm laser beam and with a thermal camera monitoring the upper surface of the suspension (Figure 3A). Figure 3B shows thermal images obtained upon illumination at three different power densities, demonstrating the heating abilities of the assemblies. The beads were further concentrated and redispersed in 200 µL (resulting concentration 0.1 wt.%) of cell culture medium, to perform the four heating and cooling cycles shown in Figure 3C, in which the illumination laser was switched on and off. This experiment confirms the reproducibility of the achieved temperature. The heating efficiency of the hybrid beads ranged between 17% and 25%, depending on the nanostar loading. This decrease in heating efficiency, as compared to the gold nanostars alone, was due to light scattering from the beads, which reduced the transparency of the sample. However, the final achieved temperature will depend mainly on the number of nanostars present under the beam and their spatial distribution.

The emission of CaF_2_:Nd^3+^,Y^3+^ was measured after incorporating the nanoparticles onto the assemblies and dispersed in cell culture media (Figure 3D, black line). A comparison of the spectrum to that from dispersed, bare CaF_2_:Nd^3+^,Y^3+^ nanoparticles in water (Figure 3D, green line) shows that, when the nanoparticles are included in the hybrid beads, an additional emission band is observed at longer wavelengths (1070 to 1150 nm). This band was found to correspond to Raman scattering from water (the Raman spectrum of pure water is also shown in Figure 3D, orange line). Raman scattering is typically less intense than luminescence, but it should be noted that here a large number of water molecules can contribute to the band. In addition, the signal from water molecules in close proximity to Au nanostars may be enhanced when excited at the plasmon resonance wavelength due to the surface-enhanced Raman scattering (SERS) effect 34, 37. Having identified the origin of this band, as well as its shape, we can now deconvolute it from the measured spectra because it is weakly dependent on temperature within the overlapping region 38, 39. Also, for the sake of accuracy, we narrowed down the wavelength range in which the Nd^3+^ emission band was integrated, to further avoid interferences from the Raman scattering band of water (I_2_ is the integrated area between 1014 nm and 1057 nm, I_1_ is the area between 1057 nm and 1074 nm). Additional details regarding the protocol followed to analyze the recorded spectra can be found in the SI, Section B3i.

### Tracking hybrid beads in 3D *in vitro* tumor models

As shown above, in addition to the excitation power, several environmental parameters can affect photothermal efficiency (e.g. heat dissipation of the environment, beads concentration). A realistic picture of the actual capabilities of the hybrid structures and their potential performance to monitor photothermal therapy requires their incorporation into representative cell models. We selected 3D tumor spheroids, as they better represent the real physiological and environmental features of the tumor, as compared to 2D cell monolayers, including the penetration of particles within the tumor and the resistance of cells to thermal shock 8. Since our multifunctional assemblies can be tracked via luminescence imaging, it should be possible to obtain information on the distribution of the probes inside the 3D model.

Spheroids were prepared using the U-87MG human glioblastoma cell line. Our protocol uses a starting number of ca. 5000 cells/spheroid in 200 µL of cell media (Day 0). Two days later they have become stable spheres with sizes around 500 µm. At this point the hybrid beads were added to the cell culture medium and incubated to allow internalization for 48 hours, at which point the spheroids presented a diameter of ca. 600 µm (see size and shape evolution of spheroids in the SI, Figure S8).

**Figure 4:**
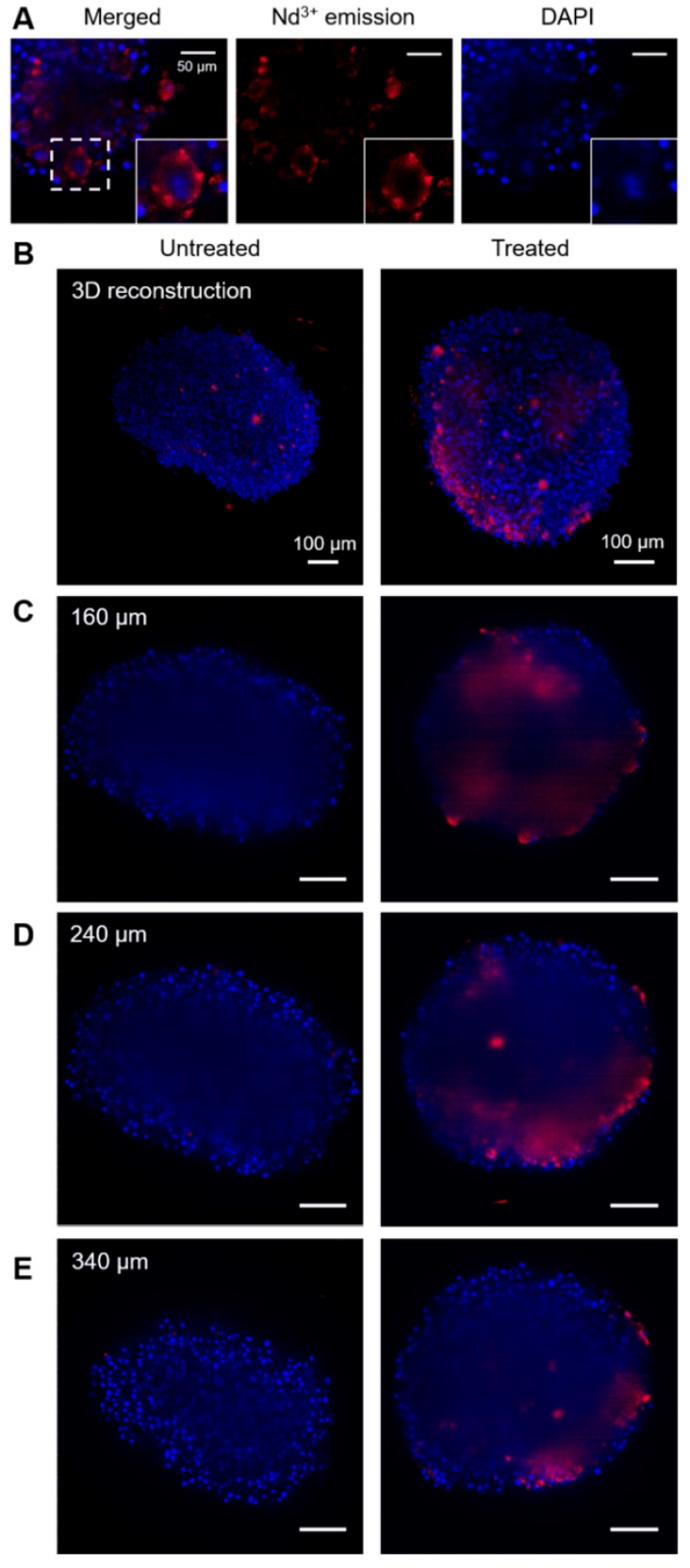
Fluorescence images of spheroids. In all images blue color stands for DAPI (cell nuclei) and red stands for Nd^3+^ emission. A) Images of the surface of a treated spheroid (150 µg/mL), showing Nd^3+^ emission apparently located in the cytoplasm. Below, two columns of images are shown, related to an untreated spheroid (left) and a spheroid treated with beads (100 µg/mL). A 3D reconstruction of the spheroid (B) and several cross-sections of each spheroid (C, D, E) are displayed.

The location of beads in cells and spheroids was monitored through Nd^3+^ luminescence, using a Lightsheet microscope modified so it can specifically record the emission band at 1050 nm, particularly advantageous to improve the penetration depth 15. Figure 4 shows a representative set of images measured for different spheroids. In these images, the red color represents the emission from Nd^3+^, while blue color depicts DAPI, which was used to label cell nuclei. Through high magnification images in the area close to the edges of a spheroid, where a better spatial resolution can be obtained, it is observed that both signals are not co-localized. Indeed, Nd^3+^ emission is observed surrounding DAPI emission, which points towards the location of the beads in the cell cytoplasm (Figure 4A).

Regarding bead distribution within the whole spheroid, Figure 4B-E shows representative images of the full structures. First, a 3D reconstruction built by stacking around 500 cross-sections is presented (Figure 4B), followed by cross sections at different depths of the same spheroids (Figure 4C-E). In every case, the image in the left column corresponds to a non-treated spheroid and is shown as a reference, whereas the spheroid in the right column was treated with 100 µg/mL of hybrid beads. All images were taken under exactly the same conditions for a fair comparison. In order to interpret the images, it must be taken into account that DAPI emits in the visible range, while Nd^3+^ does in the near-infrared, within the second biological window. Accordingly, it is expected that the signal from DAPI is partially lost due to absorption when it has to travel through the spheroid, while the Nd^3+^ signal should remain largely unaltered 15. It should be noted that, the reference spheroid (left column) does not display any signal in the Nd^3+^ luminescence range inside the spheroid (above 850 nm). We do observe however some NIR emitting objects outside of the untreated spheroid, which are commonly seen in all samples and can be assigned to small fibers present in PBS or agar, used to mount the samples. As they mainly appear outside the spheroids and present a distinctive shape, these features can be easily identified. Accordingly, we can confirm that the observed NIR luminescence inside treated spheroids stems solely from Nd^3+^, and that the hybrid beads are entering the spheroids down to at least 200 μm. It is worth noting that the spatial resolution and intensity of DAPI luminescence is partly lost inside the spheroid due to scattering and absorption along the light path. Luminescence from Nd^3+^ is also affected, especially in terms of spatial resolution. However, its emission intensity can be clearly detected inside the spheroid, likely because the selected wavelength matches the second biological window.

**Figure 5:**
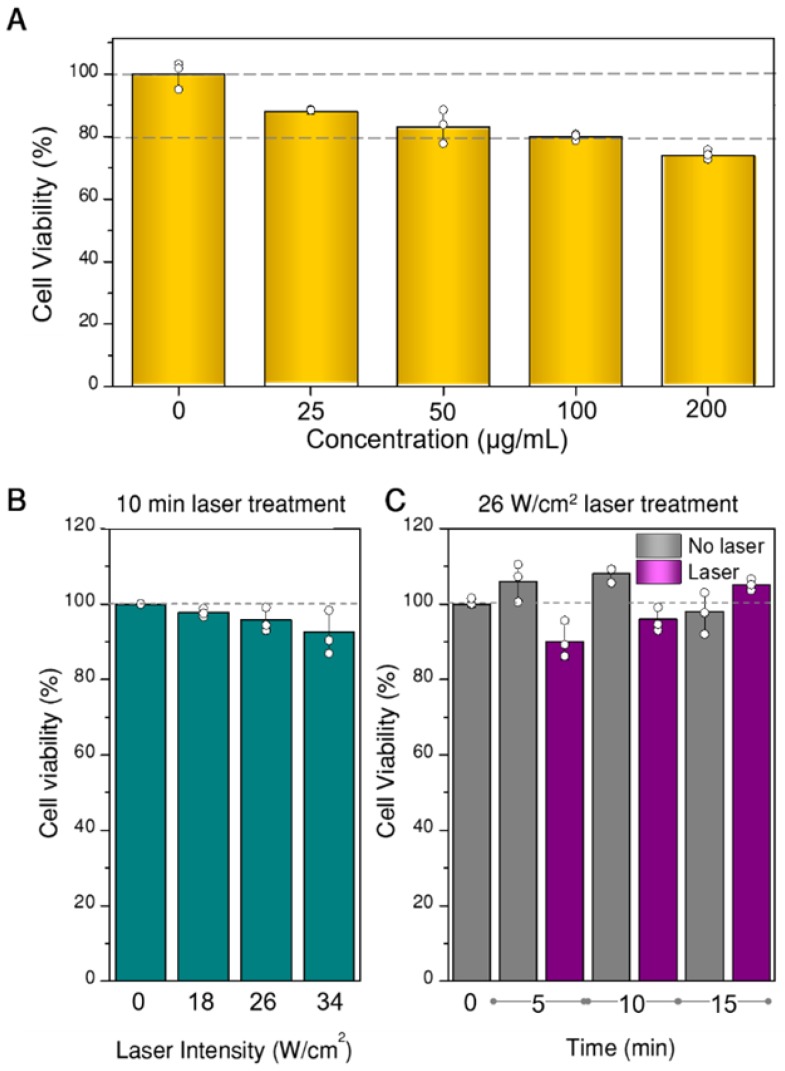
Viability of spheroids as a function of the concentration of hybrid beads in cell culture media (A), of laser power density at 808 nm for 10 min illumination (B), and of illumination time at 26 W/cm^2^ power density (C). All experiments were done in triplicate, data are shown as mean and standard deviation.

Prior to any photothermal experiments, we studied the viability of U87MG spheroids when exposed to different concentrations of hybrid beads, to determine the most suitable concentration range. For these experiments, the CellTiter-glo 3D test (Promega), which uses ATP as cell viability indicator, was applied. The results are plotted in Figure 5A, in which the concentrations refer to hybrid beads diluted in EMEM (200 µL). However, we determined by inductively coupled plasma-mass spectrometry (ICP-MS) measurements that only 2-8% of the beads were actually internalized in the spheroids. Such a low internalization efficiency is likely due to a large percentage of beads not even coming into contact with the spheroid, which lies at the center of the culture well. Still, minor toxicity is observed in all samples, from which 100 µg/mL was determined as the upper limit to be used. Regarding the initial proportion of living and non-living cells in the spheroid prior to any photothermal treatment, we also analyzed the possible existence of a necrotic core, as often happens in 3D cell models 40. In our case, necrotic cores were not identified within the working period (see SI, Figure S10). Finally, aiming at setting the parameters for photothermal treatment (time and laser power), we tested the viability of spheroids at different illumination powers and times, in the absence of beads (Figure 5B,C). We found that the effect of the laser in the selected power range is negligible within the treatment time (10 min). The observed variability, up to 15 min, was also within experimental error.

### Thermometer pre-calibration

With all this information at hand, we can now start designing photothermal treatments while simultaneously monitoring temperature. However, accurate temperature readings require recalibration of ln(I_2_/I_1_) vs. T 33, 41. In eq. (1), ΔE is independent of the external environment due to electrical shielding of 4*f* orbitals. However, B is a correction parameter related to detected differences in the emission intensities, and thus it is only constant for a fixed experimental scenario. Indeed, changes may arise from the selection of light detector, or the presence of a potential absorber in the light path toward the detector 42. This event is likely to occur in practical therapies, as light will travel through non-transparent media, such as cells, blood, culture media, etc. In our case, gold nanostars will also play a role as their absorbance at the emission wavelength cannot be neglected (see Figure 1A).

To overcome this hurdle, thermal measurements should be preceded by a recalibration step to determine ln(B) under the exact experimental conditions. This preliminary step has been formerly done by measuring the emission from the nanoparticles at different illumination powers 33, 41. Since heaters are present, each power will produce a distinct temperature, following a linear dependence. At zero power the system is at room temperature, which is known, so we can safely determine ln(B) through eq. (1) by fitting ln(I_2_/I_1_) data recorded at different powers to a straight line.

**Figure 6:**
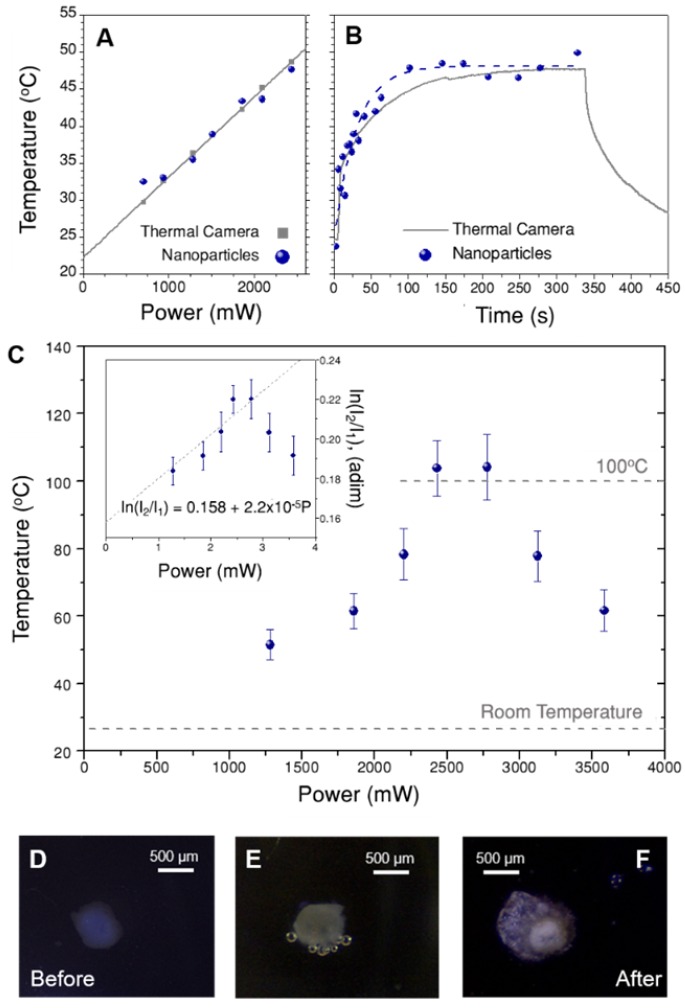
A) Temperature obtained for a solution of beads illuminated at different powers, measured both with a thermal camera (grey squares) and by nanothermometry (blue dots). B) Heating curve of the dispersion of beads over time. At time zero, the laser was switched on at 2400 mW. The dashed blue line is an exponential fit to the data. Note that figures A and B have the same vertical scale. C) Temperature measured at different excitation powers in a spheroid treated with hybrid beads (70 µg/mL), calculated by nanothermometry. The inset shows the same data as intensity ratio, i.e., prior to applying the calibration. Three representative images of the spheroid are shown below, to show its appearance, D) before the treatment, E) after 3 min illumination at 2500 mW (35 W/cm^2^) and F) at the end of the treatment. Please note that the spheroid may move and rotate due to the thermal gradient created in the well.

We first tested the protocol in the dispersions of beads shown in Figure 3C, which can also be accurately monitored with the thermal camera. Following the explanations above, the initial step comprised measuring luminescence spectra at different illumination powers and processing the spectra to obtain ln(I_2_/I_1_) values. These values were then used to recalibrate ln(B), which was found to be 0.443 in this case (Figure S13A). Introducing this number in eq. (1) we can now calculate the temperature that corresponds to each ln(I_2_/I_1_). The obtained results are shown in Figure 6A, together with the temperature recorded by the thermal camera. Both thermal measurements were found to be in agreement, with differences typically below 1.5 ºC, which validates the protocol.

It is now possible to apply this method to investigate the thermal evolution of the solutions after switching on the illumination, i.e. starting at room temperature, until thermal equilibrium was reached. Figure 6B presents the thermal data over time obtained through nanothermometry (blue dots) and with the thermal camera (grey line). As expected, temperature follows an exponential growth until reaching thermal equilibrium, in both cases at 48 ºC. Indeed, the trend is largely the same, regardless of the method used to measure temperature. The only difference is that equilibrium is reached slightly earlier according to the nanothermometers (see exponential fit, i.e. the dashed blue line). Definitive conclusions on the origin of this slight discrepancy would require a better accuracy than that achieved here. However, we hypothesize that the differences between both techniques play a role, likely because nanothermometers are always located close to the gold nanostars, while the thermal camera measures the average temperature in the whole area covered by each pixel.

To apply this recalibration method to 3D cell models, the set-up in Figure 3A was kept the same; only the solution of beads was replaced by a spheroid immersed in cell medium (EMEM). As a consequence, the thermal camera only records the temperature on the surface of EMEM, and it cannot directly monitor the spheroid itself. Recalibration of ln(B) was first performed using a starting sacrificial tumor spheroid, which was illuminated at different laser powers. The obtained values of ln(I_2_/I_1_) vs. power are plotted in the inset of Figure 6C, as the average of three different spectra, with error bars representing the corresponding standard deviations. In principle, we expected that the intensity ratio would increase linearly with the illumination power, due to photothermal heating. This is confirmed in Figure 6C, but only for the lower excitation powers considered. However, for laser powers above 2700 mW an apparent thermal decrease was found. Using these first data to recalibrate ln(B), a new value of 0.444 was obtained, i.e. slightly below the one measured for CaF_3_:Nd^3+^,Y^3+^ in water, and almost equal to that obtained for beads in EMEM (further details on this calculation are given in Section B3ii and Figure S14 of the SI). This result suggests that the change in ln(B) is mainly due to gold nanostars and EMEM, and not so much to the presence of cells. This new ln(B) value was used to calculate the corresponding temperatures in the full power range, as shown in the main graph of Figure 6C.

According to these results, the temperature increases up to 104 ºC, then reaches a plateau and decreases at powers above 2700 mW (38 W/cm^2^). This behavior is consistent with a water-based environment like the cell culture medium, in which evaporation starts consuming energy at around 100 ºC. The subsequent decrease of temperature might be related to two simultaneous effects. The first one is the potential reshaping of gold nanostars 43, whose tips have been shown to become more rounded at high temperatures, thereby triggering a blue-shift of the near-infrared plasmon resonance. This would have detrimental effects, both on the absorbance of the sample at 808 nm and on its heating efficiency. Absorbance measurements of gold nanostars treated at 100 ºC (Figure S15) confirmed that after 10 minutes the plasmon shift was around 15 nm, and absorbance at 808 nm decreased by 6%. As the experiment in Figure 6C was done keeping each laser power for 3 min, the actual time at this temperature is shorter than that. Accordingly, reshaping might only be a partial contribution to the temperature decrease. The second possible reason is the reduction of collective heating effects 44, 45, which are probably strong here. In other words, the spheroid may undergo expansion and fracture, resulting in a reduction in the density of hybrid beads. As the final temperature is the sum of the contributions by all heaters, this morphological change results in a delocalization of the hot area and a temperature decrease around the nanothermometers. These findings were supported by visual observations during the experiment, with the aid of a portable microscope. Three selected images are shown in Figure 6D-F, in which two principal effects are observed: creation of bubbles when the medium started evaporating (Figure 6E), and expansion and fracture of the spheroid after complete irradiation (Figure 6F).

The described protocol yields temperature values that are consistent with the effects observed on the spheroids. However, it may follow from this discussion that a recalibration of ln(B) would be needed each time any parameter is changed in the sample or its environment, thus requiring a new sacrificial spheroid. To avoid that, we tested an alternative strategy based on the dependence of temperature with time during illumination. It is clear from Figure 6B that temperature increases exponentially over time, as dictated by thermal equilibration equations 46. At sufficiently short time points, this trend can be approximated to a straight line. The proposed time-based strategy comprises recording emission spectra within the starting time range and fitting the obtained values to a straight line. This fit is related to ln(B) in the same way as the power trend in Figures 6A and C, assuming room temperature at time zero (See section B3ii of the SI and Figure S13B for a detailed discussion on the method). The main limitation here is related to our time resolution during the experiments (between 30 and 50 s for the spheroids), which limits the number of points we can use in the fit. However, using two spheroids, identical to that in Figure 6C, to test this new strategy, we estimate that the time-based protocol yields differences in ln(B) below 2% (exact obtained values are 0.443 and 0.451). It can thus be considered a fair approximation, and we can use it in subsequent photothermal experiments, avoiding the need for extra samples for recalibration. Further details on ln(B) calculations are given in the SI (Section B3ii and Figure S14).

### Photothermia and *in situ* temperature determination

In order to study the local temperature in tumor spheroids during illumination and the related cell survival rate, four sets of samples were prepared. Each set was treated with a different concentration of hybrid beads: 70 µg/mL, 43 µg/mL, 20 µg/mL and no heaters (CaF_2_:Nd^3+^,Y^3+^ nanoparticles alone were added to the latter set to locally measure temperature). During the experiments, two different excitation powers, 26 W/cm^2^ and 31 W/cm^2^, were used and each experiment was carried out in triplicate, to determine the statistical error. In every case, the treatment time was fixed to 10 min.

**Figure 7:**
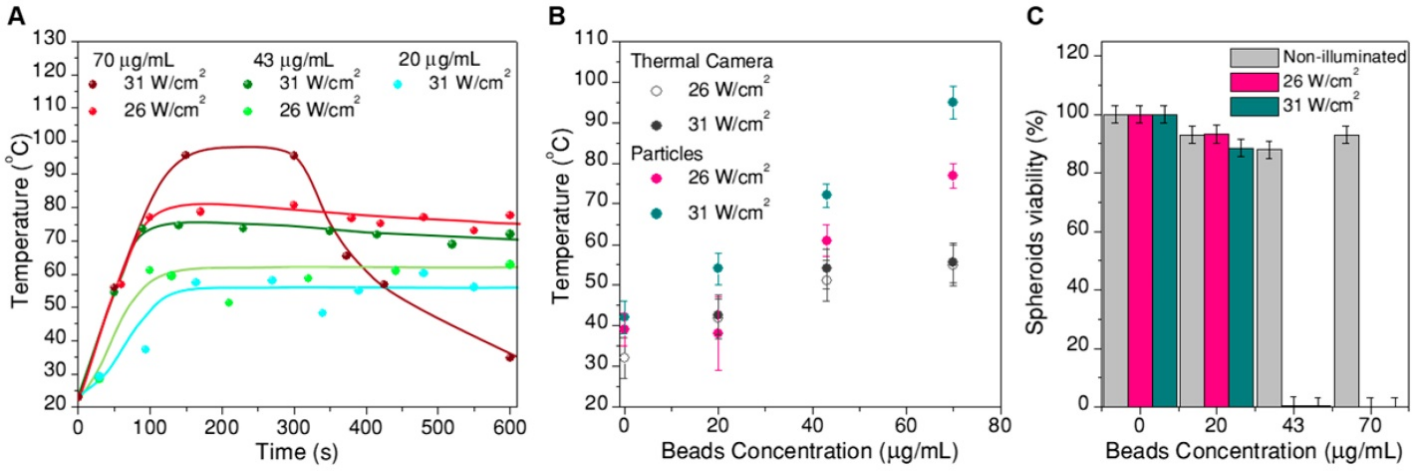
A) Temperature measurements inside spheroids during photothermal treatment: thermal evolution over time, for treatments at different excitation powers and concentrations of hybrid beads. B) Average temperature at thermal equilibrium for each treatment, compared to the temperature determined by a thermal camera. C) Viability of the spheroids at 24h after photothermal treatment

**Figure 8:**
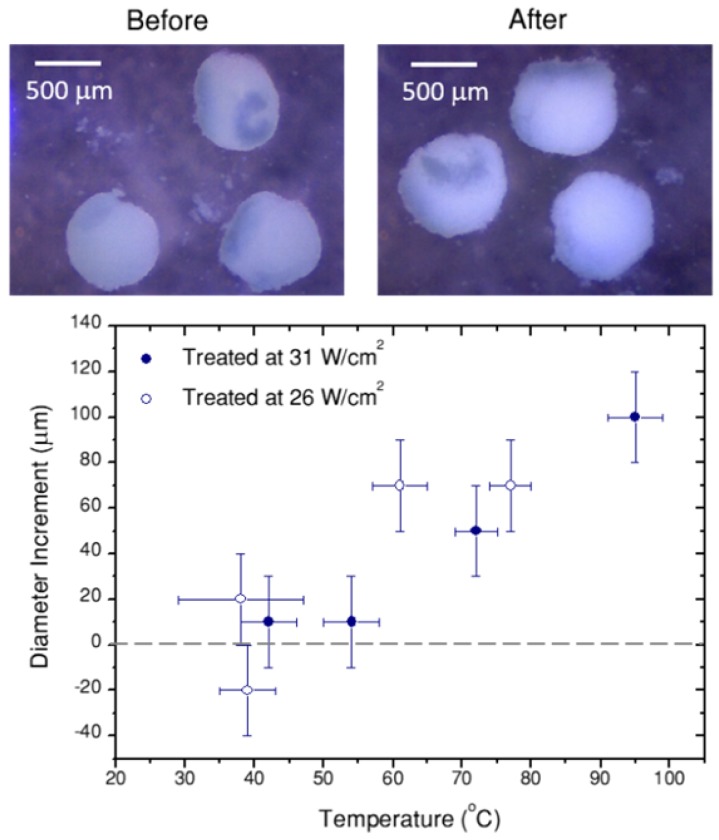
Portable optical microscope pictures of spheroids before and after laser treatment (10 min, 26 W/cm^2^, 70 µg/mL hybrid beads). Note that probably due to thermal currents, spheroids move during the treatment. In the graph, the diameter difference before and after the treatment are heating is plotted versus the temperature achieved in the treatment. Error bars correspond to the thermal resolution and the standard deviation of size.

The recorded thermal data after transforming intensity ratios into temperature are plotted in Figure 7. The time evolution of temperature (Figure 7A) consistently follows an initial fast increase until thermal equilibrium is reached at around 100s. These data were used to determine ln(B). After this time, temperature remained stable for most of the samples. The only deviation was found for spheroids treated under the most extreme conditions (70 µg/mL, 31 W/cm^2^, wine-colored data in Figure 7A), at which the boiling point of water was likely reached prior to cooling due to expansion of the spheroids after treatment. To a lesser extent, some expansion was also observed for spheroids reaching temperatures above 55 ºC (Figure 8). In the time range at which thermal equilibrium was reached (above ⁓100s), the observed thermal fluctuations can be considered as a measurement of the thermal resolution for each experiment. Since thermal resolution is limited by the signal-to-noise ratio in the spectra, it depends again on excitation power and beads concentration. We found typical values between 3 ºC and 4 ºC, but it could be as high as 9 ºC for the sample with the lowest concentration of beads and the lowest excitation power. For the sake of clarity we did not include such strongly fluctuating data in Figure 7A, and considered this experiment as a limiting situation for the validity of the thermometry method. These results can be found in the SI (Figure S16).

Plotted in Figure 7B is the average temperature within the equilibrium state, where the error bars are given by the thermal resolution (in this case the lowest concentration, lowest excitation experiment is included). The data clearly show that the reached temperature consistently increases with the concentration of beads and with laser power, as expected. We also included in this plot the temperature measured by a thermal camera (with its corresponding 5 ºC accuracy, as given by the commercial brand). The temperature measured by the particles is consistently higher than that measured by the camera, in agreement with the sensitivity of the particles toward local temperature in the spheroid, while the camera monitors the temperature at the surface of the cell medium. This comparison provides a clear idea on the importance of considering local heating for this type of applications.

Finally, Figure 7C shows the cell viability in the spheroid, 24h after photothermal treatment, related to the respective non-treated reference. Correlating these data with the thermal information in Figure 7A,B, we can conclude that local temperatures between 40 ºC and 55 ºC have little or no effect, for a 10 min treatment. Instead, as soon as 55 ºC is surpassed, cells in the spheroid are almost completely dead (viability below 1% for a treatment reaching 61±4 ºC, viabilities below 0.3% consistently determined above 70 ºC. It should be noted that a ±3% statistical error was obtained). This result shows a sharp temperature dependence of cell viability in spheroids, clearly defining a life/death threshold, which in our case was set around 55 ºC. These results are in agreement with previous reports 8 on the viability of PC3 prostate cancer spheroids, in which the authors determined that after similar treatment times the threshold temperature was between 51 ºC and 53 ºC. This difference might be related to the observed swelling of the spheroid, which happens within the same temperature range (Figure 8). Such a size increase has also been observed in earlier works devoted to hyperthermia treatment, and was ascribed to expansion of the intracellular space 47.

## Conclusions and perspective

A hybrid heater/thermometer nanoprobe was designed, comprising gold nanostars and CaF_2_:Nd^3+^,Y^3+^ luminescent nanoparticles on a colloidal carrier. Both nanoparticle elements can be excited at the same wavelength, within the first biological window (808 nm), whereas nanothermometers emit within the second biological window (1050 nm). We showed that near-IR emission can be used to track the position of the beads within cell models, by means of a commercial Lightsheet microscope. The same irradiation conditions were subsequently used to measure absolute temperatures *in-situ* inside 3D tumor models. The obtained thermal data were consistently linked to perceptible events occurring to the spheroids, such as bubbling at around 100 ºC, or swelling at temperatures where necrosis was expected. Accurate thermal readings required a pre-calibration step at the same experimental scenario in which the photothermal treatment was to be performed. We determined that this step can be reduced to a linear fit of emission data recorded at short times (<100 s in our experiments), even if only few data could be recorded within this time frame. The time resolution of our thermal experiments was around 30 s, and thermal resolution typically around 4 ºC (though it may vary for different experiments).

This work sets the path toward *in vivo* experiments, but this step would require certain improvements to optimize the hybrid probes e.g. reducing bead size. If novel designs are to be proposed, we would particularly point towards strategies that increase the number of nanothermometers per gold nanostars, to favor their luminescence intensity and reduce time resolution. It should be stressed that gold nanostars have to be slightly (>5 nm) separated from the luminescent nanoparticles to avoid luminescence quenching. Special attention should also be paid to chemical strategies that provide robust probes and allow surface functionalization. This is particularly important on the way towards clinical applications, to favor the internalization and circulation time of the probes. Also, along the path of clinical translation, illumination doses need to be reduced. In this regard, the present work should be taken as a proof-of-concept, as the volume of the clinical samples and the commonly deep-seated location of tumors within the body must be considered when designing appropriate illuminating and imaging devices, which would definitively affect the required power range.

## Materials and Methods

### Materials

Hydrochloric acid (HCl, 37%) was purchased from Panreac. Hydrogen tetrachloroaurate trihydrate (HAuCl_4_・3H_2_O, ≥99.9%), sodium citrate tribasic dihydrate (≥98%), silver nitrate (AgNO3, ≥99%), L-ascorbic acid (AA, ≥99%) were purchased from Sigma-Aldrich and O-[2-(3- mercaptopropionylamino)ethyl]-O'methylpolyethylene glycol (PEG-SH, Mw 5,000 g/mol) was purchased from RAPpolymere. Amino functionalized polystyrene beads (NP size 483 ± 3 nm) were purchased from IKERLAT Polymers (Lasarte, Spain). All glassware was washed with aqua regia, rinsed 3-fold with milli-Q water and dried before use. Milli-Q water (resistivity 18.2 MΩ·cm at 25 °C) was used in all experiments.

For the synthesis of lanthanide-doped CaF_2_ the starting reagents were calcium chloride (≥ 99%, Sigma-Aldrich) and ammonium fluoride (98+%, Acros) as CaF_2_ precursors, neodymium chloride (99.9%, Alpha Aesar) and yttrium chloride (99.99%, Alpha Aesar) as lanthanide sources, and sodium citrate tribasic dihydrate (Sigma-Aldrich) as a surfactant. All chemicals were used without further purification, and all experiments were carried out using nanopure water.

### Synthesis of gold nanostars

Au nanostars (AuNS) were prepared by a modified seed-mediated growth method to show a plasmon resonance peak at around 800 nm 31. Briefly, the seed solution was prepared by adding 0.5 mL of a 1 % citrate solution to 9.5 mL of boiling 0.5 mM HAuCl_4_ solution under vigorous stirring. After 15 min of boiling, the solution was cooled down to room temperature and then kept at 4 °C for long-term storage. The as-synthesized Au nanoparticle seeds had an LSPR maximum at 519 nm. For Au NS synthesis, 50 μL of the citrate-stabilized seed solution was added to 1 mL of HAuCl_4_ (0.25 mM) solution containing 2 μL of HCl (1.0 M) in a 2 mL glass vial at room temperature, under moderate stirring. Quickly, 10 μL of AgNO_3_ (3 mM) and 5 μL of ascorbic acid (100 mM) were added simultaneously to the above solution. The solution rapidly turned from light red to greenish indicating the formation of AuNSs. Immediately after synthesis, the solution was stirred with PEG5000-SH (15 molecules/nm^2^) for 15 min, washed by centrifugation (6000 rpm, 45 min, 10 °C) and redispersed in water. The used solution had an absorption coefficient of 0.4 cm^-1^ at 400 nm.

### Synthesis of Ln^3+^-doped nanoparticles

CaF_2_ doped with a 1% of Nd^3+^ and 15% Yb^3+^ were prepared by a hydrothermal method 32. The concentration of dopants was selected based on a previous work 33, and is [Nd^3+^] = 1 mol% and [Y^3+^] = 15 mol%. The mass of chloride salts containing calcium, neodymium and yttrium was calculated to obtain 3.5 mmol of the final material, being 90 mg NdCl_3_, 160 mg YCl_3_ and 432 mg CaCl_2_. Chloride salts were first dissolved in 10 mL of water, and stirred for 30 min. Then, 5.8 g of sodium citrate in 10 mL of water was added to the solution, and stirred overnight. The resulting milky solution turned transparent upon addition of 7 mL of water containing 324 mg of NH_4_F. After further vigorous stirring for 30 min, the solution was introduced in the Teflon insert (50 mL) of a pressure digestion vessel (DAB-2, Berghof). The reactor was then introduced in an oven and kept at 180 ºC for 5 h. After this time, the synthesized nanoparticles were recovered from the solution by centrifugation (5500 rpm, 12 min), and then redispersed in water, sonicated, and washed twice by centrifugation. The particles were kept in water solution at 1.3 mg/mL or 0.7 mg/mL of Nd^3+^ concentration (measured through ICP).

### Assembly of gold nanostars and Ln^3+^-doped nanoparticles to polystyrene beads

0.5mL of PEG-stabilized AuNS solution 0.1 mM or 0.5 mM was added dropwise onto 100 µL of PS beads (0.25 % in solid in 0.1 M NaCl) in an Eppendorf tube under sonication during 5 min and then, 0.5 mL of Ln^3+^-doped NPs (0.1 M NaCl solution) was added and sonicated 5 min and the particle mixture was incubated overnight. Two different Ln^3+^-doped nanoparticles concentrations (1.3 mg/mL or 0.7 mg/mL of Nd^3+^) were used to obtain different luminescence loading. The unbound nanoparticles were removed by two centrifugation steps (2000 rpm, 4 min, 20 °C) and the samples were redispersed in Milli-Q water. TEM images of hybrid beads individually modified with each type of nanoparticle, as well as of the full structure, are shown in Figure S3. Figure S4 shows several images of the beads loaded with different concentrations of nanoparticles. In Figure S5 we show the absorbance spectra of the gold nanostars alone and in the final beads. The main difference observed in the lower wavelength range is due to scattering by the beads, meaning that the plasmonic resonance of the stars is not strongly modified during the synthesis, which would happen if their shape is changed or if they aggregate.

### Silica coating AuNSs/CaF_2_:Nd^3+^,Y^3+^ immobilized polystyrene beads

Silica coating of the assembled beads was carried out by diluting 200 μL of an aqueous dispersion of beads in a mixture of 1 mL of water and 1.8 mL of isopropanol. With this solution in a 5 mL glass vial and under stirring, 18 μL of ammonia (32%) was added, to set the pH between 9 and 10. After further stirring over 30 min, 50 μL of additional CaF_2_:Nd^3+^,Y^3+^ (0.3 w/w %) was added, to enhance the luminescent character of the assembly. The solution was then sonicated for ten minutes, and stirred for 20 more minutes. Afterwards, 200 μL of TEOS solution in isopropanol (2 w/w %) was added. The mix was left stirring overnight, and finally, the particles were washed by centrifugation three-fold (2000 rpm, 10 min) to recover the resulting silica coated beads that can be redispersed either in water or in cell media. In addition to the images shown in Figure 2C and D, representative images of the coated beads are included in Figure S7.

The concentration of each type of particle in the beads was measured through ICP, both with the hybrid beads dispersed in water and in the cell media (EMEM). The CaF_2_:Au ratio in the final assembly was determined to be 20:1 in mass (though it varies between samples and can be as low as 10:1 or as high as 30:1, depending on the starting concentrations used). This means that there are around 700 CaF_2_ nanoparticles per gold nanostar, which makes sense given the small volume of CaF_2_ nanoparticles compared to that of gold nanostars.

### 3D cell culture

Experiments in cells were performed using U-87MG (epithelial, human glioblastoma cancer) as a model cell line. The cells were cultured in EMEM cell media completed with a 10% fetal bovine serum (FBS) and 1% antibiotics (penicillin and streptomycin, PS), all purchased from Invitrogen. To prepare spheroids, the selected number of starting cells (either 5000 or 10000) were plated in the well of an ultra-low attachment U-bottom 96-well plate (Nexcelom bioscience) in 200 μL of complete cell media. The cells naturally group at the center of the well, forming a rounded cluster after 24h, which becomes a compact spheroid after 48h (day 2), and can then be easily handled. The size and shape evolution of the spheroids is shown in Figure S8.

To treat the spheroids, a concentrated solution of hybrid beads in water was diluted in EMEM (with 10% FBS) to reach the desired concentration. The final volume of water was always kept below 25% of the total volume, as we determined that the effect of water on the viability of spheroids was not detrimental at that concentration. The spheroids were then cultured in 200 uL of this solution. This step was always done after 60h (at the end of day 2) of life of the spheroid. The spheroids were incubated like that for 48h to let the particles internalize before doing any further experiment.

### Spheroid fluorescence imaging: Lightsheet microscope

#### Preparation of spheroids for imaging

In order to image spheroids in the Lightsheet microscope, they need to be fixed, stained and mounted in agar matrix. The followed protocol started with washing the spheroids by keeping them in PBS for 10 min while gentle shaking was applied. This process was repeated three times. They were then fixed by immersing them in a solution of paraformaldehyde (2% in PBS). The spheroids were kept in this solution for 2 hours, in which they stayed on a rocking shaker at 4 ^o^C. Afterwards, the spheroids where washed again in PBS, this time the process was repeated three times in 5 min cycles. Once the spheroids were fixed, they were stained with DAPI. In order to do so, they were kept in a 16 μM solution of DAPI in PBS. This step was done at room temperature, in complete darkness and for 40 min. Then, the spheroids where washed in PBS twice, 10 min per cycle. The spheroids where then mounted in low melting agarose to be imaged, paying attention to the temperature of the agarose, that was 35 ^o^C when the spheroids where added to it.

When required, propidium iodide (PI) staining was performed before fixation, but after the first washing cycles in PBS. In order to do so, spheroids where immersed for 20 min in a 1 mg/mL solution of PI in PBS. After this process, spheroids where washed twice in PBS, and the preparation protocol for Lightsheet microscopy continued as explained above.

#### Imaging technique

To image cell spheroids, a Lightsheet microscope (Zeiss, Lightsheet Z.1) was used. DAPI fluorescence was excited at 405 nm and its emission detected using a bandpass filter (420 - 470 nm). To use the luminescence of Nd^3+^ to locate the particles, Nd^3+^ was excited with the 561 nm laser line of the microscope and its luminescence was recorded in the near-infrared range. In order to remove any autofluorescence background, the microscope was manipulated to incorporate a longpass filter (Thorlabs, cut-off 850 nm).

Propidium iodide (PI) has been occasionally applied to study the proportion of live and dead cells in spheroids. Its use was restricted in most experiments, since the luminescent signal of this dye creates a background that hinders the signal from Nd^3+^. However, we judged important to understand the composition of the spheroids in terms of cell life before proceeding with the experiments (Figure S10).

### Routine characterization techniques

Transmission electron microscopy (TEM) images were obtained in a JEOL JEM-2100F electron microscope, at an acceleration voltage of 120 kV. Samples for TEM analysis were prepared by adding a single drop (2 µL) of the aqueous solution (ca. 0.1 mg/mL in milliQ water) of particles onto a copper grid coated with a carbon film (Electron Microscopy Sciences). Further details on the preparation of grids can be found in the SI, Section A4.

UV-Vis spectra were measured in an Agilent 8453 UV-Vis diode-array spectrophotometer. Zeta potential measurements were performed in a Malvern Zetasizer 3000 HS particle size analyzer (Malvern Instruments, UK). Fluorescence measurements were carried out with a PerkinElmer LS55 Fluorescence Spectrometer. ICP-MS measurements were performed on a Thermo iCAP Q ICP-MS (Thermo Fisher Scientific GmbH, Bremen, Germany). A ASX560 autosampler was coupled to the ICP-MS (CETAC Tech, Omaha, NE, USA). In order to digest the different materials for ICP experiments, 100 µL of CaF_2_ particles aqueous solution was digested overnight in 10 mL HNO_3_ (65%, Scharlau) at 70 ^o^C. Full beads were digested overnight in aqua regia. Spheroids were digested in aqua regia as well, but they required sonication before and after a 24h digestion time.

### Optical Set-up and Hyperthermia Experiments

Optical characterization of the emission of CaF_2_:Nd^3+^,Y^3+^ nanoparticles was done by illuminating the sample at 808 nm with a fiber-coupled laser diode (Lumics, LU808T040), with the beam collimated to a 3 mm diameter spot. Emission was collected by a lens, filtered (longpass filter, Thorlabs, cut-off 850 nm) to remove any laser signal, and then recorded by an InGaAs spectrometer (Sol 1.7, B&WTek).

Hyperthermia experiments were performed in the same way, keeping the spheroids in a 200 μL well with quartz bottom (to avoid any luminescent signal from the well) that was illuminated from top (Figure 3A). Laser light was not blocked just after the well, but two centimeters thereby, avoiding any possible heating due to light absorbance by the materials constituting the set-up. During the experiments, spheroids were kept in the well with 180 μL of cell media, and were moved to the incubator just after the treatment. Aiming to obtain further information from the spheroids and the effect of the treatment, a portable microscope (Aven, Mighty Scope 1.3M, 175x) was used to acquire images of the different samples before and immediately after the treatments.

To study the viability of the cells in spheroids CellTiter-glo 3D test (Promega) was used, always 24h after the treatment.

### Raman spectra

The Raman spectra of water was recorded using a Renishaw inVia Raman Reflex microscope.

## Supplementary Material

Supplementary figures and tables.
