# Supplementary material for: Thermal monitoring during photothermia: hybrid probes for simultaneous plasmonic heating and near-infrared optical nanothermometry
Source: Theranostics. 2019 Sep 25;9(24):7298–312. doi: 10.7150/thno.38091 (PMC6831289; doi:10.7150/thno.38091)
Supplement: Supplementary file 1 — Supplementary figures and tables. [file thnov09p7298s1.pdf]

SUPPLEMENTARY INFORMATION OF

Thermal Monitoring during Photothermia: Hybrid Probes for Plasmonic Heating and Near-Infrared Optical Nanothermometry

Marta Quintanilla[1], Isabel García[1], Irene de Lázaro [2], Rafaela Paz García[1,2], Malou Henriksen-Lacey[1], Sandra Vranic[2], Kostas Kostarelos[2], Luis M. Liz-Marzán[1, 3]

[1] Bionanoplasmonics Laboratory, CIC biomaGUNE and CIBER-BBN, Paseo de Miramón 182, 20014 Donostia-San Sebastián, Spain

[2] NanomedicineLab, Faculty of Biology, Medicine & Health, The University of Manchester, Manchester M13 9PT, UK

[3]Ikerbasque, Basque Foundation for Science, 48013 Bilbao, Spain

Contents

A. CHARACTERIZATION OF PARTICLES 2

1. Heating Efficiency of Gold Nanostars 2
2. Dynamic Light Scattering and Zeta Potential of the Assemblies 3
3. Assembly of Gold Nanostars and Ln^{3+}-Doped Nanoparticles to Polystyrene Beads. 4
4. TEM Grids Preparation 5
5. Silica Coating of AuNSs/CaF_2:Nd^{3+},Y^{3+} Immobilized Polystyrene Beads 6

B. PREPARATION AND CHARACTERIZATION OF 3D CELL MODELS 7

1. Size and Shape Evolution of Spheroids 7
2. Cell Viability of Spheroids before Photothermal Treatment 7
3. Methods to Measure Temperature in Spheroids 9
   i. Determination of the Intensity Ratio 9
   ii. Resetting lnB 11
4. Stability of Gold Nanostars at 100 ºC 14
5. Low Concentration, Low Power Measurements 15

C. REFERENCES 16

A. CHARACTERIZATION OF PARTICLES

1. Heating Efficiency of Gold Nanostars

In order to determine the light-to-heat conversion of the gold nanostars used in this paper, we used the method developed by Roper et al. [1] In this protocol, we evenly illuminated 1mL of gold nanostars, dispersed in water, with a laser at 808 nm (the wavelength to be used all along this work). The temperature at the surface of water was monitored over time with a thermal camera (FLIR A35). When the temperature reached thermal equilibrium, the illumination was turn off, and the cooling down curve was recorded, since it can be used to quantify heat dissipation to the environment.

The heating efficiency, η, is then given by the formula:

$$\eta = 100x \frac{Q_p - Q_w}{P(1-10^{-OD})} \; ; Q_i = \frac{mC_p}{\tau} \Delta T \qquad \text{(S1)}$$

where P is the laser power and OD the optical density of the sample as measured. The denominator provides information on the energy that the particles are interacting with. Q_i are the heat dissipation terms of the particles (i = p) and of the solvent (water, i = w), which are defined by the mass of suspension (m), the isobaric heat capacity of the solvent (c_p), the characteristic time of the exponential cooling-down curve, τ, and the thermal increment when thermal equilibrium is reached, ΔT.

For the sample illuminated with a laser power of 520 mW, in the conditions of our experiment, we obtained τ = 384 ± 10 s (the error is the standard deviation for two different measurements; an exponential fit of the cooling-down curve is shown in Figure S1 as an example). By measuring two different samples at different concentrations, we obtained a value for the heating efficiency of 74.0 ± 0.3 %.

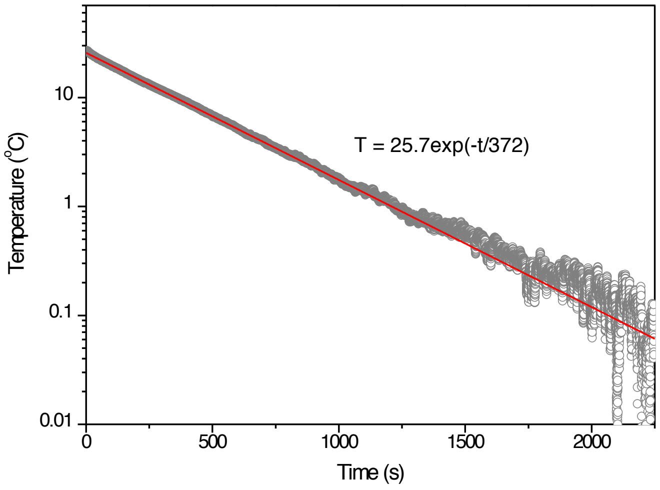

Figure S1. Cooling-down curve in a logarithmic plot, used to calculate the heating efficiency of gold nanostars. The red line represents an exponential fit.

2. Dynamic Light Scattering and Zeta Potential of the Assemblies

The assemblies were characterized after each step through dynamic light scattering (DLS) and zeta potential, obtaining the data shown in Figure S2. The starting gold nanostars present a zeta-potential of -25mV, and the CaF_2 nanoparticles around -7 mV. The starting beads present a positive zeta potential of 40 mV. Accordingly, adsorption by electrostatic interactions was expected.

After assembling the three components, the final zeta-potential was -18 mV, which is consistent with the beads being coated by the nanoparticles. These nanostructures were finally coated with silica and reached a zeta potential of -40 mV.

Regarding the hydrodynamic diameter, DLS was used as a routine screening method. The starting beads presented a hydrodynamic diameter of 515 ± 6 nm (standard deviation after three measurements), with a polydispersity index (PDI) of 0.38. Once gold nanostars and CaF_2 were attached to their surface, the hydrodynamic diameter reached 750 ± 20 nm, and the PDI became 0.78. This increase in PDI is consistent with a decrease in the size homogeneity of the assemblies, as not all of them present exactly the same nanoparticle loading. After coating the assemblies with silica, the hydrodynamic diameter further increased to reach 830 ± 30 nm, while the PDI increased to 0.85.

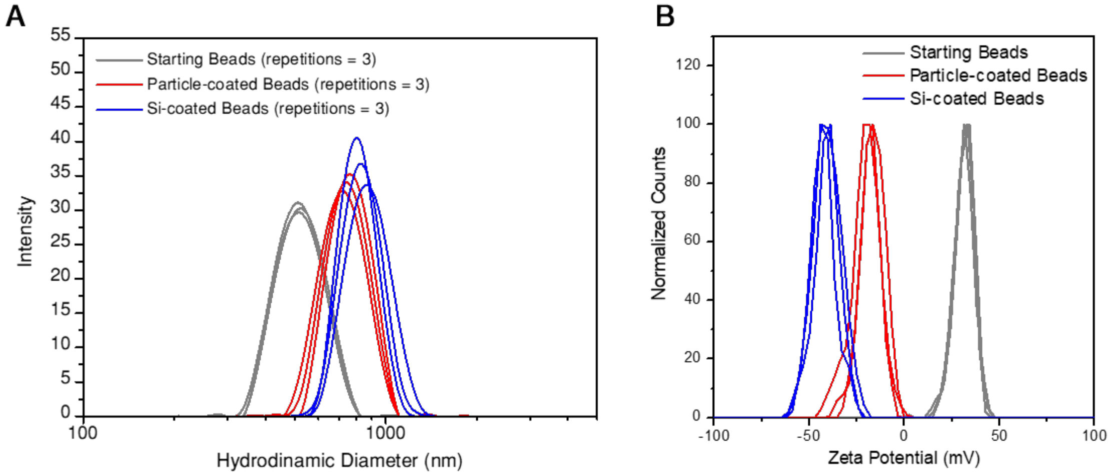

Figure S2. DLS and zeta-potential measured for the starting beads, the beads coated with particles, and the silica-coated assemblies.

3. Assembly of Gold Nanostars and Ln^{3+}-Doped Nanoparticles to Polystyrene Beads

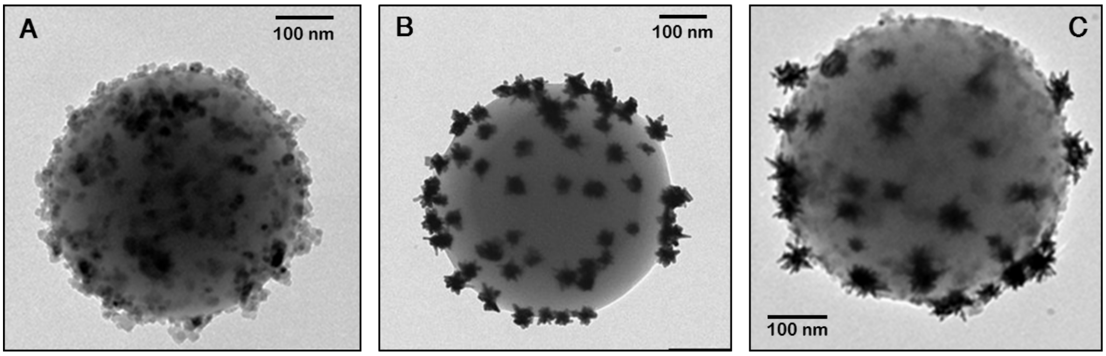

Figure S3. TEM images of PS assembled with Ln^{3+}-doped NPs (A), Au NSs (B) and Au NSs and Ln^{3+}-doped NPs (C).

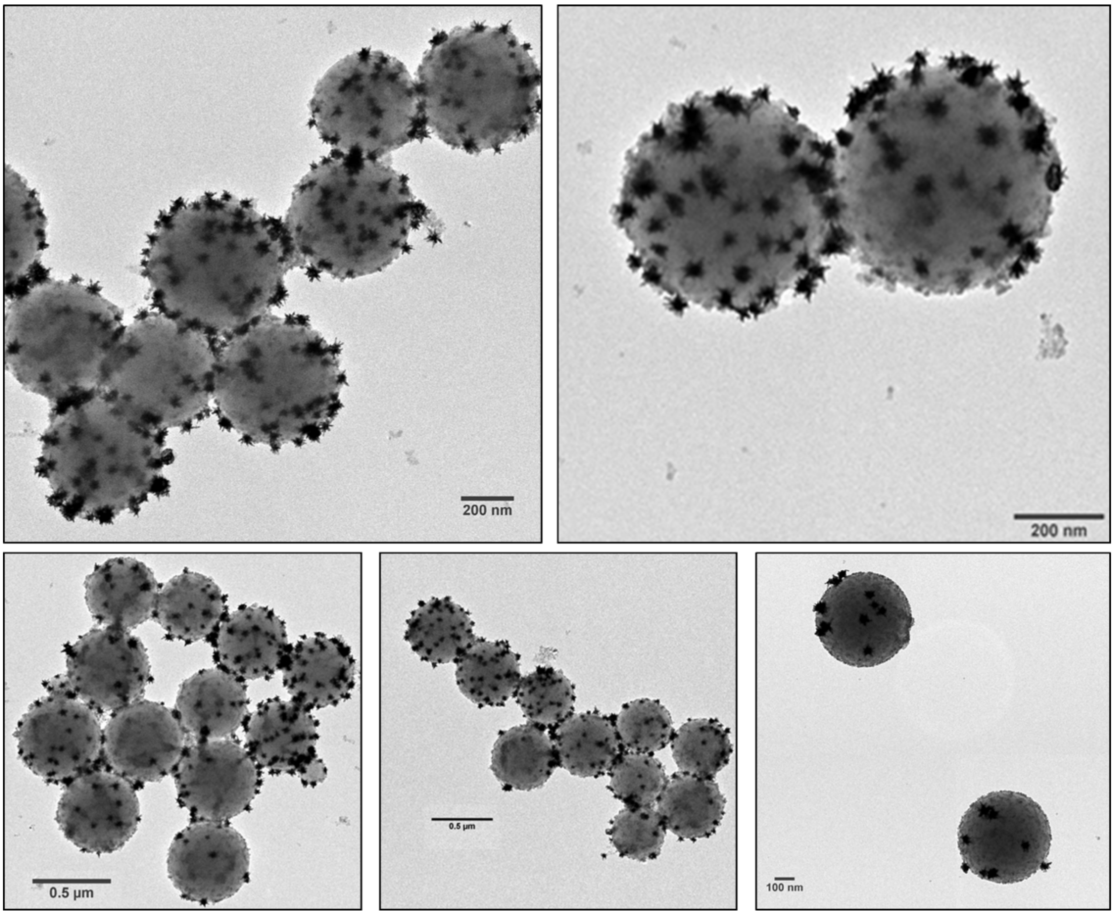

Figure S4. PS assembled with AuNSs and Ln^{3+}-doped NPs at different loadings.

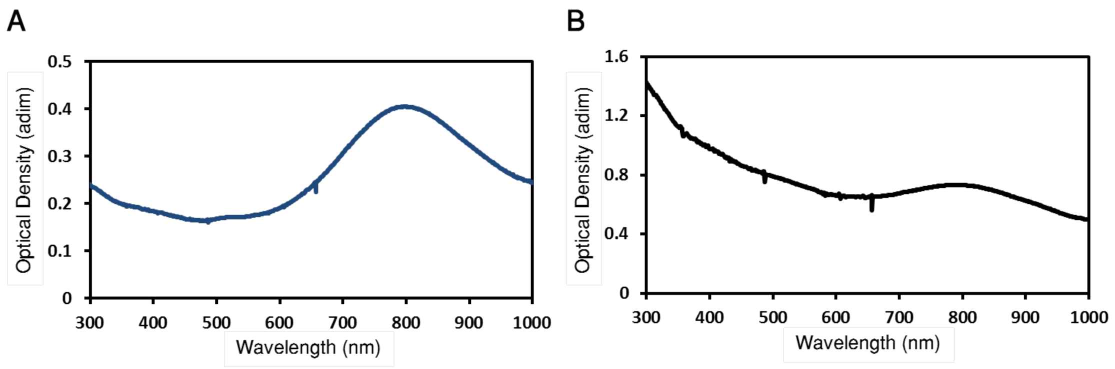

Figure S5. UV-Vis-NIR spectra of AuNSs before the assembly process (A) and of polystyrene assembled with AuNSs and luminescent NPs (B).

4. TEM Grids Preparation

Typically, we prepared standard carbon-coated copper grids for TEM simply by letting dry a drop of a concentrated bead solution on top of them. However, in the case of the uncoated assemblies we observed that the drying protocol strongly influenced the appearance of the assemblies. If too fast, some proportion of gold nanostars and CaF_2 nanoparticles were found around the beads (Figure S7 shows two extreme cases), but not linked to them. We found out that the fraction of free particles could be strongly reduced by simply keeping the grids in a controlled humidity environment, to slow down the drying process, or by dipping the grid in a dilute solution of beads and then taking it out slowly. This was the case of the images shown in Figure S3 and S4 and Figures 2C and D of the main text. This extra care was not required in any case for silica coated beads.

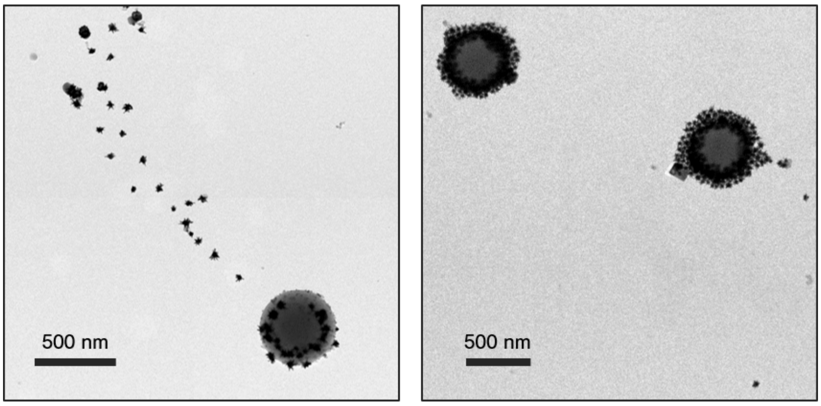

Figure S6. TEM images of assemblies prepared letting the grids dry fast.

5. Silica Coating of AuNSs/CaF_2:Nd^{3+},Y^{3+} Immobilized Polystyrene Beads

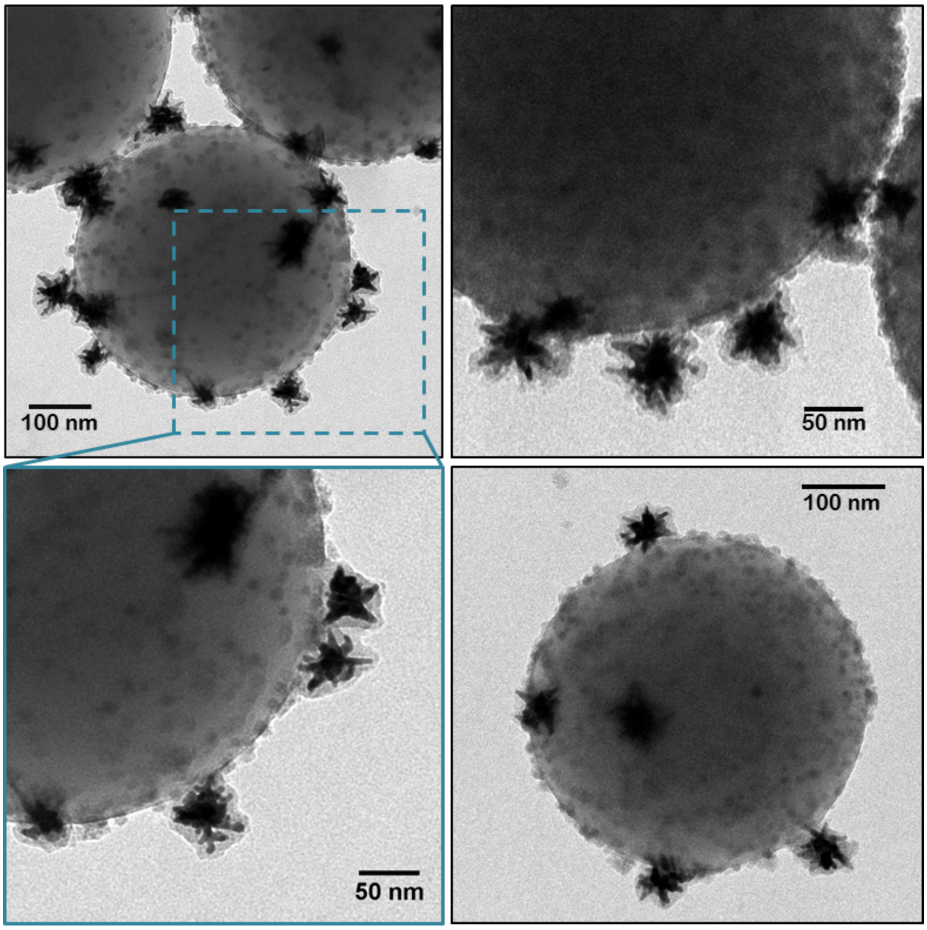

Figure S7. TEM images of silica-coated polystyrene beads assembled with AuNSs and CaF_2 nanoparticles.

B. PREPARATION AND CHARACTERIZATION OF 3D CELL MODELS

1. Size and Shape Evolution of Spheroids

Following the culture protocol described in the main paper, the size dispersion of the different spheroids is small (as measured using ImageJ analysis). Indeed, the error bars in Figure S8 stand for the standard deviation obtained after measuring the diameter of 15 to 20 spheroids. Besides, it can be seen in the pictures of Figure S8 that the spheroids show a good sphericity. It is worth mentioning that whenever a spheroid did not have a suitable shape (they may start as two differentiated spheres that usually merge after two days, but would not become as rounded as the standard ones), they were then discarded for photothermal treatments as they might be detrimental for the reproducibility of the experiments.

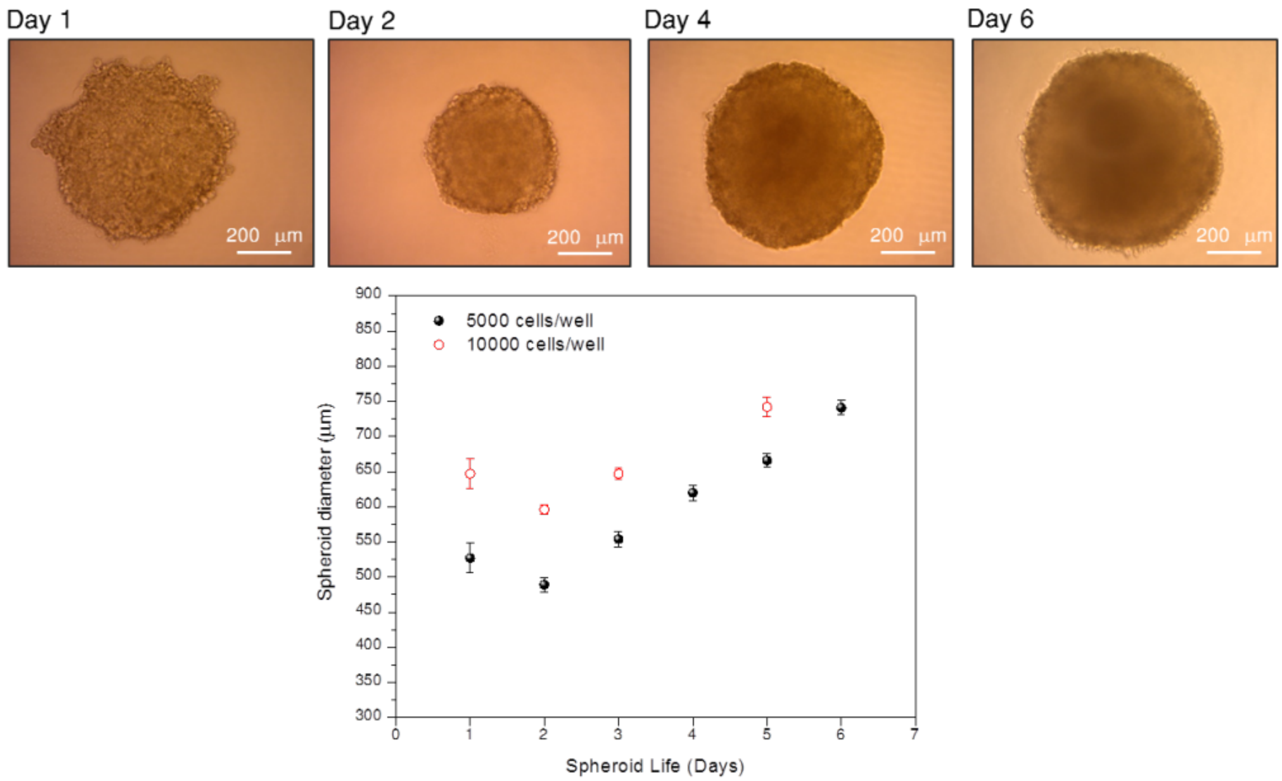

Figure S8. Size and shape development of U-87MG spheroids. The obtained measurements are the average values obtained after measuring 12 different spheroids on different days, adding fresh media every 24 h.

2. Cell Viability of Spheroids before Photothermal Treatment

It is worth mentioning that 3D cell spheroids may comprise a core composed of dead cells, due to their limited access to nutrients and debris externalization [2]. Such a core is relevant here, since those cells would interact differently with the nanoparticles, and cannot be affected by photothermia. Following these thoughts, we studied the status of the spheroids on the third day after planting the cells, i.e. matching with the time at which particles should be internalized, and just before photothermal treatment.

In Figure S10A optical microscopy images of spheroids at different growth time-points are shown. In addition to the images of U-87MG spheroids, we added for comparison purposes images prepared with the MCF-7 cell line (human breast, adenocarcinoma), following exactly the same preparation protocol as explained above. The images clearly show a dark area at the center of MCF7 spheroids that does not appear so clearly in the U-87MG cell line, and may correspond to the dead core.

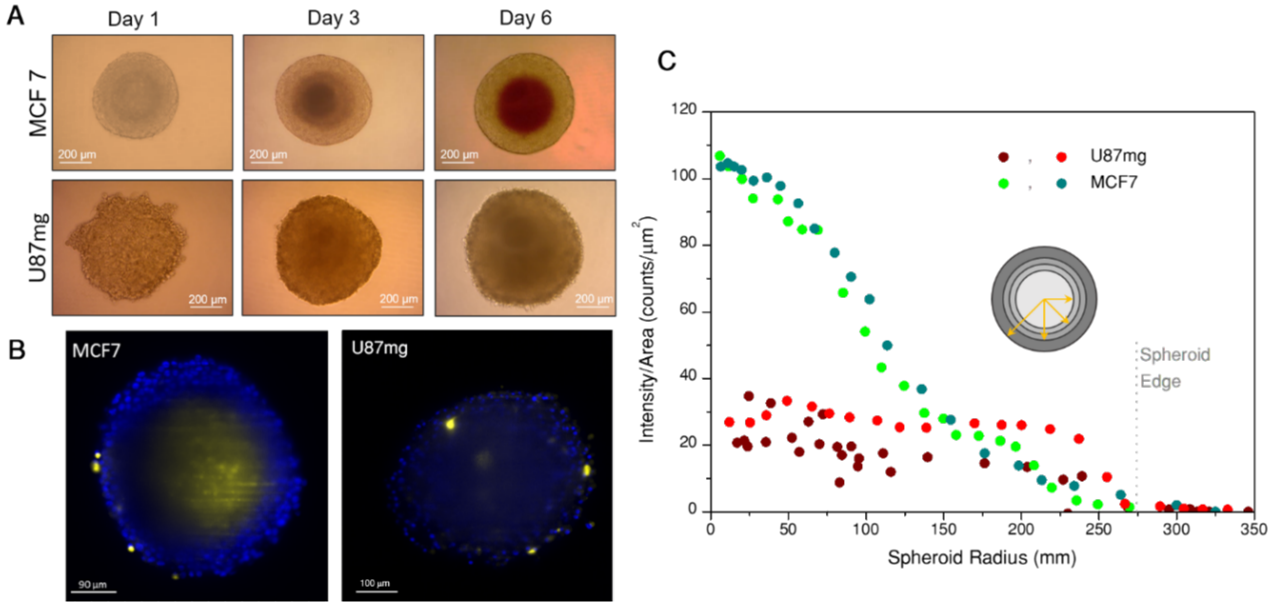

Figure S10. Comparison between two 3D spheroids, one prepared with the MCF-7 cell line, and one with the U-87MG line. A) Optical microscopy images of spheroids at different time points. B) fluorescence images of a cross-section of the spheroids on day 3. In these pictures the blue color stands for DAPI and the yellow color for PI. C) Intensity of PI signal per unit of area in concentric crown-shaped areas of the spheroid. Two spheroids of each type are shown.

Two spheroids of each cell line were imaged in a Lightsheet microscope on day three, after staining them with PI and DAPI. A representative fluorescence image of each cell line is shown in Figure S10B, with PI labelled in yellow and DAPI in blue. They correspond to a centered cross-section of the spheroid (246 μm depth). Both images were recorded and treated in exactly the same way, so they can be fairly compared. The images show that, while the MCF-7 cell line presents a distinct yellow core, in U-87MG spheroids only a few cells stain positive with PI. This suggests the existence of a dead core in MCF-7 spheroids, which is not found in U-87MG spheroids at this time-point. For further clarity, in Figure S10C we show the integrated PI emission intensity in concentric ring-shaped areas using ImageJ analysis. In U-87MG spheroids, the intensity per unit of area is almost constant in the whole spheroid, whereas in the case of MCF-7 spheroids, it continuously grows as we go deeper into the spheroids. This suggests that MCF-7 spheroids present a necrotic core after three days of growth but U-87MG spheroid cores remain viable. This characteristic life/death status of U-87MG spheroids supports their use in this work, as it facilitates the observation of any thermally-triggered death effect.

3. Methods to Measure Temperature in Spheroids

i. Determination of the Intensity Ratio

In the proposed nanothermometry approach, the emission of the $^4F_{3/2}$ state from Nd^{3+} at around 1050 nm is used to determine the temperature, and the corresponding luminescence is excited at 808 nm. Temperature is specifically inferred from the intensity ratio between the emissions (intensity calculated through integration of the emission band) of two sub-levels, the splitting wavelength being 1057nm. This value was set following the spectroscopy study presented in a previous work [3].

When the luminescent nanoparticles are part of the hybrid probe, the luminescent spectrum of Nd^{3+} is expected to remain unaltered, since lanthanides are weakly affected by the environment. However, as explained in the main text, in the measured spectra we see a new shoulder with the maximum intensity at around 1110 nm (see figure 3D of the main paper). It has been determined that this band corresponds to the Raman modes of water excited at the laser wavelength, whose intensity can be enhanced due to the presence of plasmonic nanoparticles. Figure S11A presents the recorded spectrum at two different laser powers. Note that the scale here has been transformed from wavelength to spectral shift, using 808 nm as the reference. In this representation the band at around 2700 cm^{-1} is Nd^{3+} luminescence, and the band at around 3300 cm^{-1} is the Raman spectrum of H_2O. Spectra were normalized to the intensity of the Raman mode, to show that their dependence on laser power (or temperature) is different. The fact that both signals overlap may constitute a source of inaccuracy when temperature is estimated, and thus this needs to be carefully discussed.

The Raman spectrum of water is, to a large extent, well known, including its dependence with temperature [4, 5]. Following these works, the spectra can be decomposed in five modes related to stretching of clusters of water molecules linked through hydrogen bonding. The Raman spectrum of water at room temperature measured in our setup is shown in the inset of Figure S11A, fitted by five Gaussian curves. The obtained modes are centered at 3115 cm^{-1} (I), 3223 cm^{-1} (II), 3403 cm^{-1} (III), 3554 cm^{-1} (IV) and 3626 cm^{-1} (V), in agreement with previous reports [4].

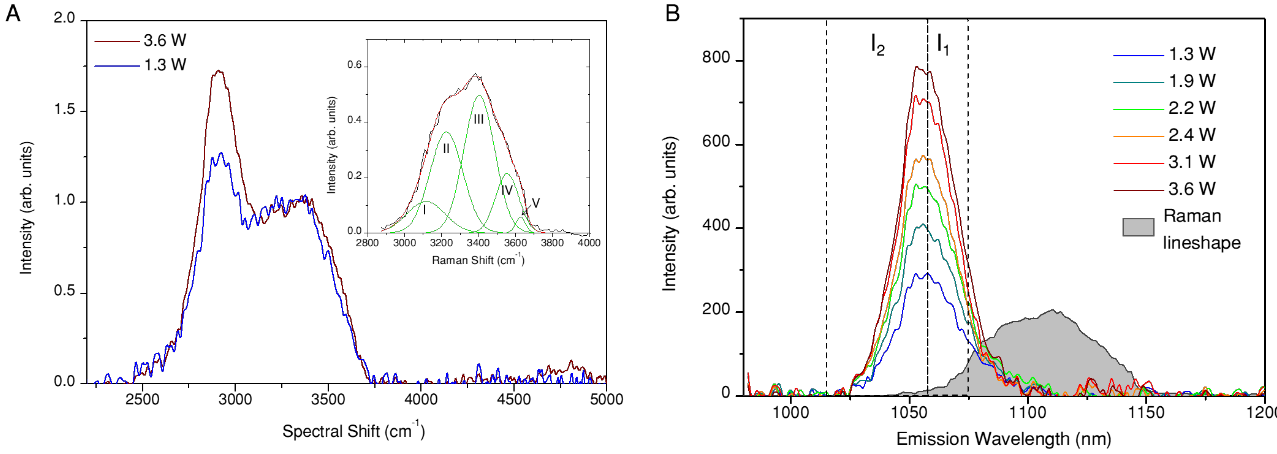

Figure S11. (A) Spectra of hybrid beads measured at two different excitation powers. The inset shows the Raman spectrum of water fitted by five Gaussians. (B) Nd^{3+} luminescence spectra measured in the hybrid probes at different powers after removing the Raman signal (plotted as a grey shadow as reference to show the overlap). I_1 and I_2 are the integrating ranges.

The main thermal differences reported for the Raman spectrum of water are the slight shift of the modes, typically patent at around 3500 cm^{-1}, and the decrease of the area of mode II compared to mode III, which typically shows up around 3200 cm^{-1}. Below 3100 cm^{-1} the effect of temperature is expected to be small, at least between room temperature and 100 ºC [4, 5]. In Figure S11A some difference can be observed in the Raman modes measured at different powers around 3500 cm^{-1}, but no differences appear clearly at 3200 cm^{-1}.
It is not possible in our case to fit the five Raman modes in every case, due to overlap with luminescence, so a fit cannot be used to deconvolute both signals. Accordingly, based on the previous observations we opted for the following protocol: first, we used the bandshape at room temperature (inset of Figure S11A) as a reference and fitted it to every recorded spectrum, adjusting its intensity. Then, the fitted spectrum was treated as part of the background, and subtracted from the main signal. In this way we obtained a series of spectra like the one shown in FigureS11B, which were then fully related to Nd^{3+} luminescence.
As mentioned above, we did not observe changes in the Raman signal below 3200 cm^{-1}. Still, to further guarantee that the Raman signal of water was not affecting our measurements, we reduced also the integration ranges used to determine I_1 and I_2. Particularly, I_2 is defined as the integrated area between 1014 and 1057 nm, while I_1 is the area integrated between 1057 and 1074 nm. In terms of Raman shift, this means that we are cutting the analysis at 3065 cm^{-1}, so it is only the tail of mode I that actually overlaps. It should be noted that for consistency, the thermal calibration of the material shown in Figure 1E of the main paper was already analyzed following these integration ranges, though the rationale behind the chosen limits is linked to these later experiments.
As described above, the Raman spectrum of water cannot be fitted by the five Gaussian curves in every case, as it overlaps with the Nd^{3+} luminescence. However, fixing modes I, II and III to the parameters found for the Raman spectrum at room temperatures, modes IV and V can be analyzed separately. The obtained results are shown in Figure S12, where their peak position (mode shift), bandwidth and area are plotted individually. Since mode V is weaker, its fit accounts for a larger error and trends cannot be set. However, mode IV shows a subtle but constant broadening, as well as a certain shift towards higher energies when power is raised. Both modifications together account for the slight differences at around 3500 cm^{-1} in the spectra shown in Figure S11A. These two changes are consistent with previous reports [4, 5] and it can be assumed that they are related to thermal differences. Instead, while the observed increase in area could be partly related to temperature as well, it can also be directly linked to excitation power, and thus, no further conclusions can be deduced from it.
In principle, Raman modes could also be used to deduce thermal information from the environment. However, here we are limited in that regard since a proper calibration versus temperature is not possible and thermal modifications are too small for the signal-to-noise ratio of the spectra. Besides, since the recorded signal is linked to the solvent, the extracted thermal information would also be linked to it, and thus the spatial resolution would be lower.

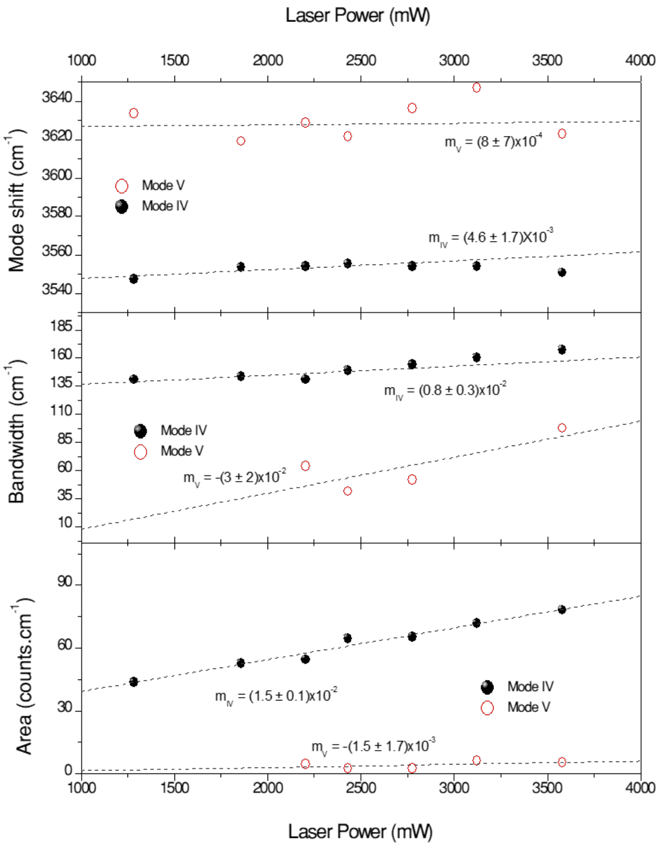

Figure S12. Parameters of the Gaussian fits obtained from the different spectra, showing the changes of modes IV and V with excitation power.

ii. Resetting lnB

As explained in the main text, temperature can be deduced from Nd^{3+} emission, through the intensity ratio of two emission bands, I_1 and I_2. This ratio depends on temperature following a Boltzmann distribution, which in logarithmic form is:

$$\ln\left(\frac{I_2}{I_1}\right) = lnB - \frac{\Delta E}{k_B T} \qquad \text{(S2)}$$

with ΔE being the energy gap between the excited states responsible for emissions I_1 and I_2, k_B Boltzmann's constant, T the absolute temperature and B an experimental constant. The emitted I_2/I_1 may change along the optical path if any component of the sample or its surroundings is not fully transparent to both wavelengths. In such a case, the calibration obtained from particles dispersed in pure water may lose accuracy and a certain recalibration would be required. Such a recalibration mainly involves resetting ln(B).

A starting strategy involves resetting ln(B) by measuring the emission from a sacrificial spheroid at different excitation powers, P. As long as the sample remains unaltered during the experiment, the relationship between temperature and power should be linear, as expressed by the point-source equation, which can be used to estimate the thermal increment created by a nanoparticle at a certain distance, r:

$$\Delta T(r) = \frac{\sigma I_0}{4\pi k_t r} \qquad \text{(S3)}$$

where I_0 is the photon flux on the particle (directly related to the illumination power), σ the nanoparticle absorption cross-section and k_t the thermal conductivity. Accordingly, following equation S2, the relationship

between $\ln(I_2/I_1)$ and power should also be linear. Then, we can assume that the extrapolated value at zero power would be $\ln(I_2/I_1)$ at room temperature, T_{RT}, since there are no additional sources of heat:

$$\ln\left(\frac{I_2}{I_1}\right) = A + \alpha P \rightarrow A = \ln\left(\frac{I_2}{I_1}\right)\Big|_{P=0} = lnB - \frac{\Delta E}{k_B T_{RT}} \tag{S4}$$

where A is the intercept and α the slope given by the linear fit (Figure S13A). This recalibration works, as shown in previous reports [3, 6]. In our case, it gives a value for lnB of 0.444, involving that the last value in Figure S13A is at 101 ºC. It could be argued that the last two values at higher powers are affected by the loss of compactness of the spheroid. If we do not consider them in the fit, lnB only changes to 0.441, which would give a highest temperature of 106 ºC. For our results here, this is not a critical difference. Still, it is illustrative to point out that, in applications requiring a finer thermal resolution, the accuracy of lnB is an important parameter to consider when designing the experiment.

This protocol based on power-dependent measurements does work, but under certain circumstances it might not be the most convenient option to follow, since recalibration should be repeated each time something changes in the experiment. In the present work this involves the requirement of new sacrificial spheroids. To avoid the use of extra spheroids, an *in-situ* alternative is to use data recorded during the starting heating trend, before thermal equilibrium is reached.

The evolution of temperature over time follows an exponential trend until reaching equilibrium at T_{max}. This can be expressed as:

$$T = T_{max} - \Delta T exp\left(-\frac{t}{\tau}\right) \ \rightarrow\ \frac{1}{T} = \left(\frac{1}{T_{max}}\right) + \left(\frac{\Delta T}{T_{max}(T_{max} - \Delta T)}\right) exp\left(-\frac{t}{\tau}\right) \tag{S5}$$

where ΔT it the thermal increment and τ the time constant. Of course, T_{max}, ΔT and τ will depend on both heat production and heat dissipation coefficients, so they will change between experiments. Since T varies exponentially, so does 1/T, as also shown in eq. (S5). Regardless of the value of the constants, since $\ln(I_2/I_1)$ depends linearly on 1/T (eq. S2), it will also show an exponential dependence with time. Moreover, at time zero (just before the illumination source is switched on) temperature has to be room temperature, which is a known value of T, and thus this point can be used to deduce ln(B).

In order to obtain the value of $\ln(I_2/I_1)$ at time zero we need to extrapolate the exponential trend to calculate the intercept. Fitting the whole exponential curve would require waiting to reach equilibrium, which during the experiment might not be convenient if we are seeking *in situ* thermal information. Alternatively, it can be assumed that, at very short times, an exponential growth (the heating-up trend) can be approximated by a straight line, given by:

$$\ln\left(\frac{I_2}{I_1}\right) = \alpha + \beta t \rightarrow \alpha = \ln\left(\frac{I_2}{I_1}\right)\Big|_{t=0} = lnB - \frac{\Delta E}{k_B T_{RT}} \tag{S6}$$

where α is the intercept and β the slope given by the fit (Figure S13B).

In Figure S13 we plotted $\ln(I_2/I_1)$ measured for solutions of beads in EMEM (presented in Figure 3 of the main paper). Figure S13A shows the power-based method. In this case ln(B) can be calculated from the intercept by adding $\Delta E/k_BT_{RT}$ (T_{RT} = 22.5 ºC), and is 0.445. In Figure S13B thermal data were recorded during the heating-up stage after switching the laser on. In this case T_{RT} was 26 ºC. Following the exponential fit (blue line), ln(B) should be calculated as y_0-A+$\Delta E/k_BT_{RT}$, where y_0 is the intercept of the fit and A the pre-exponential factor. In this case, ln(B) is calculated to be 0.445, the same as before. Finally, by applying a linear fit (dark red line), ln(B) takes again the value of 0.445. The equivalence of the three options is thus demonstrated.

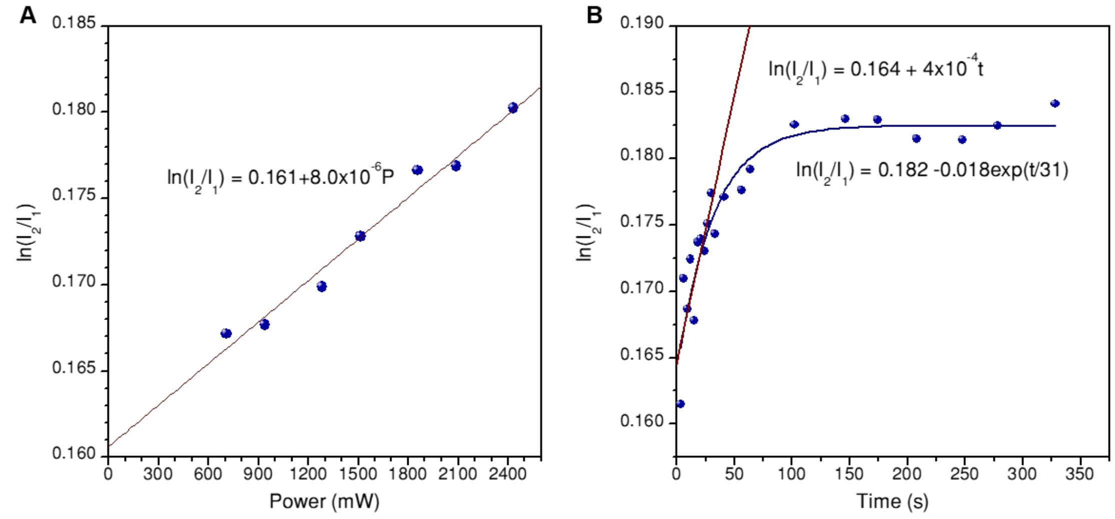

Figure S13. Methods to reset ln(B) applied to measurements in a dispersion of hybrid beads in EMEM illuminated at 808 nm: A) based on the dependency of $\ln(I_2/I_1)$ on power; B) based on the time evolution of $\ln(I_2/I_1)$. Dark red lines are linear fits to the data, while the dark blue line is an exponential fit. To deduce ln(B), room temperature is 295.6K in A and 299.1K in B, and $\Delta E/k_B$ = 84 K.

We can now apply both methods to the recalibration of ln(B) in 3D spheroids. In this case, the limitation is the time resolution of the luminescence experiment, in our case between 30 and 50 s. However, the heating trend has been observed to be slower and the linear fit can reach up to 100 s. Still, only two or three points can be measured within this time-frame. This low number of data may limit the accuracy of the strategy. However, in our case, the obtained value of ln(B) following this technique is comparable to that obtained with the power-based strategy, which validates the protocol. This is demonstrated in Figure S14. In particular, shown in Figure 14B are the results obtained in two equivalent spheroids measured with 2800 mW illumination. The power-

based technique applied to one spheroid yields a value for ln(B) of 0.444, whereas the time-based strategy applied to two spheroids gives 0.443 and 0.451.

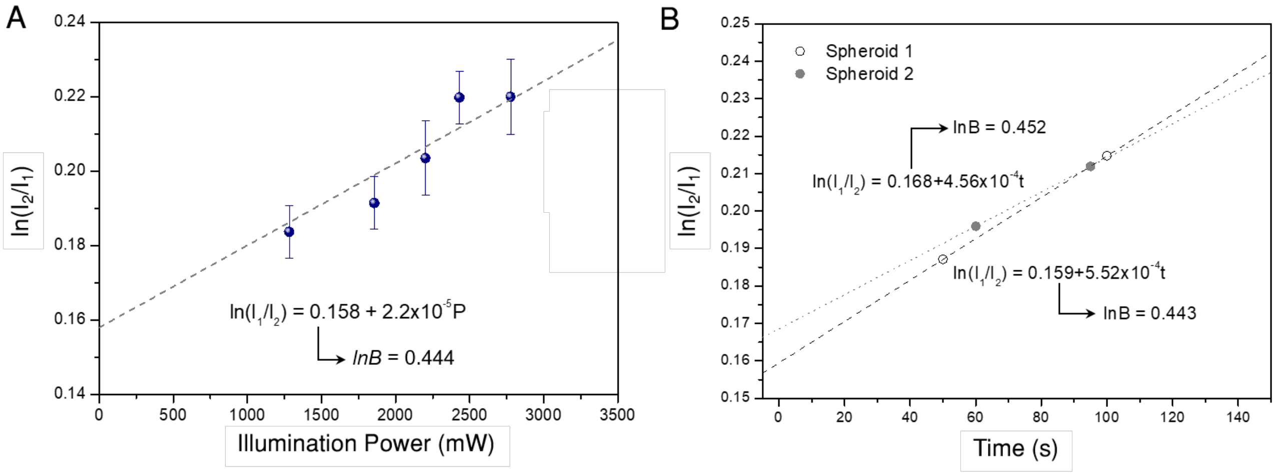

Figure S14. Methods to reset ln(B): A) based on the dependency of $\ln(I_2/I_1)$ on power and B) based on the time evolution of $\ln(I_2/I_1)$. Straight dashed lines are, in every case, fits to the data. To deduce ln(B), room temperature is 296K, and $\Delta E/k_B$ = 84 K.

4. Stability of Gold Nanostars at 100 ºC

The maximum temperature achieved in the photothermal treatments is 100 ºC. In this section the effect of this temperature on PEG-modified gold nanostars is discussed based on the absorbance of two different batches of water-dispersed particles. Absorbance spectra have been taken before any heating treatment, and then after keeping the nanoparticles at 100 ºC for 10 min (the standard treatment duration applied in this work) and for 30 min. The obtained results are shown in Figure S15. All curves have been normalized at 400 nm to consider only the amount of gold present in the solution and exclude the effect of the water volume, which may evaporate during the treatment.

It is clear in the plots that the LSPR shifts over time towards smaller wavelengths. Such a spectral blue shift is typically related to a morphological change in nanostars, whose tips become more rounded, to an extent that they may eventually become spheres [7]. In the samples studied here, after a 10 min treatment the LSPR was blue-shifted by 15 nm, which may have some effects on the heating performance of the stars, but is not critical. Indeed, in terms of absorbance there is only a ~6% decrease at the laser wavelength, 808 nm (marked with a vertical dashed line in the figure). Instead, a 30 min treatment does represent a major change in the optical properties of the nanoparticles, whose LSPR shifts by almost 100 nm and the absorbance at 808 nm is reduced by 42%.

Besides the LSPR blue shift, some aggregation can be also deduced from changes in the absorbance at around 950 nm. However, given the differences between this experiment and thermal treatments in spheroids, in terms of environment and mobility of the particles, it cannot be concluded whether this effect would also occur then.

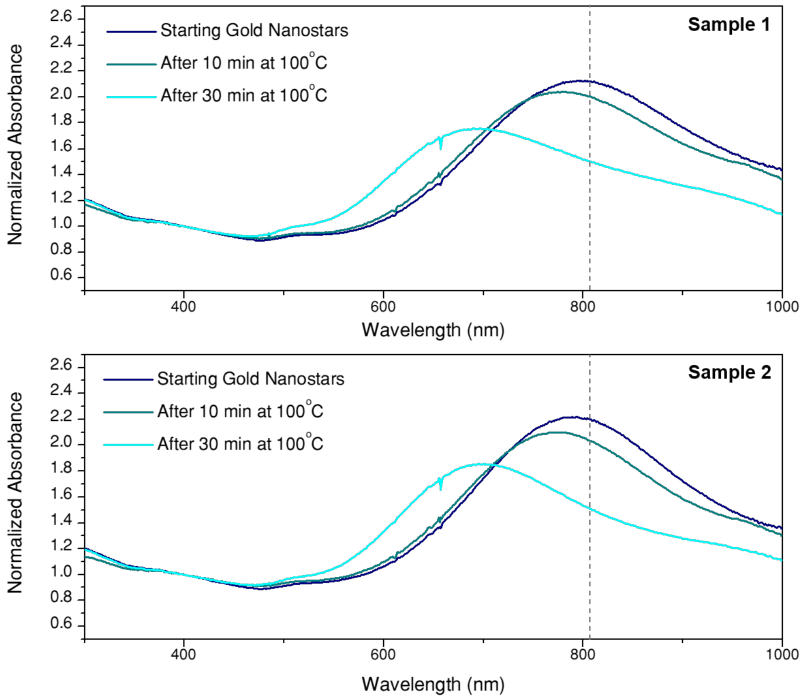

Figure S15. Absorbance of two different batches of PEG-functionalized gold nanostars, measured as synthesized (dark blue line), after treating them at 100 ºC for 10 min (dark cyan line) and after 30 min of thermal treatment (light cyan line). As the samples are dispersed in water, spectra were normalized at 400 nm to account for a possible effect of water evaporation.

5. Low Concentration, Low Power Measurements

As mentioned in the main text, the data obtained in spheroids with the lowest concentration of beads (20 µg/mL) and illuminated with the lowest excitation power (26 W/cm^2), lose accuracy due to a lower signal-to-noise ratio, and have not been discussed in the main paper. For completeness, the obtained results are shown in Figure S16.

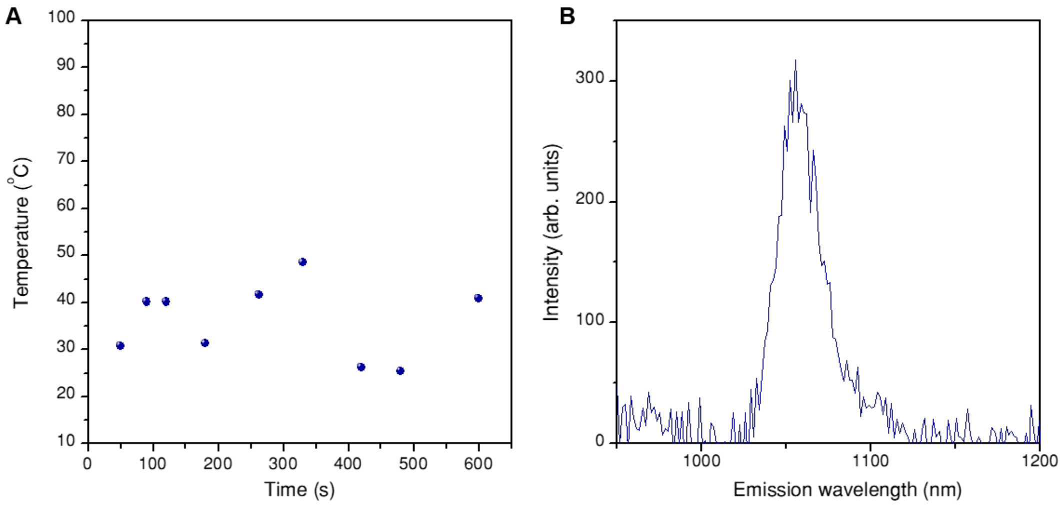

Figure S16. A) Temperature measured in spheroids treated with the lowest concentration of beads (20 µg/mL) and illuminated with the lowest excitation power (26 W/cm^2). B) Example of emission spectrum.

C. REFERENCES

1. Roper DK, Ahn W, Hoepfner M. Microscale Heat Transfer Transduced by Surface Plasmon Resonant Gold Nanoparticles. J Phys Chem C. 2007; 111: 3636-41.

2. Zanoni M, Piccinini F, Arienti C, Zamagni A, Santi S, Polico R, et al. 3D tumor spheroid models for in vitro therapeutic screening: a systematic approach to enhance the biological relevance of data obtained. Sci Rep. 2016; 6: 19103.

3. Quintanilla M, Zhang Y, Liz-Marzán LM. Subtissue Plasmonic Heating Monitored with $CaF_2:Nd^{3+},Y^{3+}$ Nanothermometers in the Second Biological Window. Chem Mater. 2018; 30: 2819-28.

4. Hu Q, Lü X, Lu W, Chen Y, Liu H. An extensive study on Raman spectra of water from 253 to 753 K at 30 MPa: A new insight into structure of water. I Mol Spectrosc. 2013; 292: 23 - 7.

5. Artlett CP, Pask HM. Optical remote sensing of water temperature using Raman spectroscopy. Opt Express. 2015; 23: 31844-56.

6. Balabhadra S, Debasu ML, Brites CDS, Ferreira RAS, Carlos LD. Upconverting Nanoparticles Working As Primary Thermometers In Different Media. J Phys Chem C. 2017; 121: 13962-8.

7. Vanrompay H, Bladt E, Albrecht W, Béché A, Zakhozheva M, Sánchez-Iglesias A, et al. 3D characterization of heat-induced morphological changes of Au nanostars by fast in situ electron tomography. Nanoscale. 2018; 10: 22792-801.
